# Development of a Novel Recombinant Full-Length IgY Monoclonal Antibody against Human Thymidine Kinase 1 for Automatic Chemiluminescence Analysis on a Sandwich Biotin-Streptavidin Platform for Early Tumour Discovery

**DOI:** 10.1155/2023/7612566

**Published:** 2023-03-17

**Authors:** Xiangbin Wang, Shan Li, Rui Zhao, Huijun Li, Peng Gao, Cuicui Jin, Cong Fang, Yongping Liu, Ailian Hei, Ji Zhou, Jin Li, Ellen He, Sven Skog

**Affiliations:** ^1^Genfine Basic Science Centre (GBSC), Floor 5, Building 33, Changping Life Valley Industrial Base, Changping District, Beijing 102299, China; ^2^Department of Medicine, Shenzhen Ellen-Sven Precision Medicine Institute, Shenzhen 518110, China; ^3^Changzhou Cancer Hospital, No. 68, Honghe Road, Xinbei District, Changzhou City, Jiangsu Province 213002, China

## Abstract

Serum thymidine kinase 1 protein (STK1p) concentration has been used successfully as a reliable proliferating serum biomarker in early tumour discovery and clinical settings. It is detected by an enhanced chemiluminescence (ECL) dot blot assay with the biotin-streptavidin (BSA) platform (a gold standard) based on chicken anti-human thymidine kinase 1 IgY polyclonal antibody (hTK1-IgY-pAb). However, ECL dot blotting is a semiautomatic method that has been limited to large-scale applications due to the differences among batches of antibodies from individual hens, and the skill level of operation technicians sometimes results in unstable STK1p values. Therefore, a highly stable recombinant chicken full-length IgY monoclonal antibody in combination with a fully automated sandwich biotin-streptavidin (sandwich-BSA) platform was developed. Hens were immunized with 31-peptide, a key sequence of human TK1 (hTK1), before constructing an immune phage display scFv library. Finally, a recombinant full-length IgY monoclonal antibody (hTK1-IgY-rmAb#5) with high-affinity binding with human recombinant TK1 (rhTK1) (3.95 × 10^−10^ mol/L), high sensitivity with hTK1 calibrators (slope of linear curve: 89.98), and high specificity with low/elevated STK1p (*r* ≈ 0.92-0.963) was identified. hTK1-IgY-rmAb#5 showed a specific immune response with thymidine kinase 1 (TK1) in TK1-positive/negative cell lysates by Western blotting and immunohistochemistry (IHC) in normal and cancer tissues. In particular, the detection of TK1 serum samples from health centres showed a high coincidence rate (*r* = 0.988, *n* = 90) between hTK1-IgY-rmAb#5 and hTK1-IgY-pAb and between the semiautomatic ECL dot blot BSA platform and the novel automatic chemiluminescence sandwich-BSA platform (*r* = 0.857, *n* = 292). hTK1-IgY-rmAb#5 is stable and highly sensitive for detecting the lowest STK1p value at 0.01 pmol/L (pM). The accuracy is high (SD < 2.5%) between different batches. It is easy to use the novel hTK1-IgY-rmAb#5 on a new automatic chemiluminescence sandwich-BSA platform. It will be beneficial for large-scale health screenings.

## 1. Introduction

Cancer is currently one of the major leading causes of human death. Highly sensitive and specific early screening assays are greatly needed to achieve the early discovery of tumours, early treatment, and early cancer recovery [1–3]. TK1, an enzyme of the pyrimidine salvage pathway, catalyses the conversion of thymidine to thymidine monophosphate, which is further converted by thymidylate kinase and nucleoside diphosphate kinase to thymidine triphosphate pools. This is an economical recycling route for the reuse of thymidine. TK1 is a key enzyme involved in DNA synthesis during the cell cycle and is thus regarded as an S-phase-specific enzyme. TK1 protein concentration and activity are closely related to the DNA synthesis rate, reflecting the proliferation rate of mammalian cells [4–6]. TK1 was found to be a precision protein molecular target for assessing the proliferation rate of growing cells in the 1960s [7]. Once multiple gene mutations occur in normal cells, they result in unlimited tumour cell growth. A noninvasive serological tumour proliferation rate detection of TK1 activity assay was considered in the clinical setting in the 1980s, and then, the isotope serum TK1 activity (STK1a) assay was improved to nonisotope technology. The BrdU-AZT-STKa kit (http://www.biovica.com) is useful, but the substrate is not specific for hTK1. Thus, infection with viral or bacterial pathogens can influence the final actual TK1 values [8]. The assay is also sensitive to pH and temperature. To date, studies using STKa assays have only been performed in oncology [9] and have focused on prognosis and monitoring treatment but not on health screening.

Since 2000, different TK1 antibodies have been used in clinical studies, such as mouse IgG monoclonal Ab [10, 11], rabbit IgG polyclonal Ab [12], and recombinant monoclonal TK1 antibody derived from rabbit [13], all of which are based on the sequence of hTK1 [6]. These antibodies are still recommended for clinical research use.

The near C-terminal of 31-peptide-^195^GQPAG PDNKE NCPVP GKPGE AVAAR KLFAPQ^225^ in human TK1 (31-peptide of hTK1) is the critical sequence for cell cycle regulation of TK1 [14] and was used to produce a mouse monoclonal antibody against a 31-peptide of hTK1 more than 20 years ago. A TK1 IHC kit (http://www.sstkbiotech.com) was used for immunohistochemistry staining in oncology, for example, in lung adenocarcinoma patients [15], cervical malignancies [16], and ovarian serous adenocarcinomas. A commercial sandwich-BSA enzyme-linked immunosorbent assay (ELISA) kit (http://www.arocell.com) was used for treatment follow-up in cancer patients [17] but not for health screening.

Based on a TK1-IgY-pAb from the egg yolk of hens immunized with 31-peptide hTK1, we successfully developed an ECL dot blot assay with the BSA platform [9]. The assay was applied not only in the clinical evaluation of prognosis, recurrence risk, and survival in cancer patients but also for early tumour screening. Based on the data of 160,086 people who underwent health examinations at four independent routine health screening centres in China from 2005 to 2018, the STK1p assay was able to distinguish between patients with benign and malignant tumours before the appearance of tumour loci in modern imaging [9].

However, differences among batches of antibodies were found from individual hens, and the limitations of semiautomatic ECL dot blotting with the BSAS platform sometimes resulted in unstable STK1p values. Although the semiautomatic ECL dot blot assay was performed with rigorous standard operating procedures (SOP) for all steps, much technical, manpower, and financial resources are needed to ensure the stable and uniform supply of high-quality TK1-IgY-pAb, limiting its wider application.

The TK1-IgY-pAb derived from chickens has more advantages as a prognostic serological biomarker compared to IgG-Ab derived from mammals in health screening [9]: (1) The advantage of chicken immunoglobulin Y (IgY) is that it is a highly conserved homologue of immunoglobulin G (IgG), and IgYs do not trigger potentially dangerous immune responses. The FC portion of the IgY-Abs did not bind to rheumatoid factor (RF), a major source of interference in many immunoassays. In contrast, RF can react with the FC portion of mammalian IgG (poly- or mAbs) in most immunoassays, thus causing false-positive results. Since RF is present in blood samples from patients and healthy individuals, nonspecific immune cross-reaction can be avoided by using hTK1-IgY-rmAb or hTK1-IgY-pAb for serological biomarker in vitro assays [18]. (2) Another interfering factor is the production of human anti-mouse IgG antibody (HAMA), which can be eliminated since chicken IgY antibody does not react with HAMA [9]. (3) Finally, chicken IgY-pAb in general shows more advantages than single antibody detection because it binds to a higher number of epitopes, yielding more accurate results. In chickens, both H- and L-chain gene loci consist of a single gene and are unable to generate antibody diversity by germline gene rearrangement, as in mammals. The diversity of the chicken antibody repertoire is mainly generated by gene hyperconversion and somatic mutation [19]. In addition, the evolutionary distance between chickens and mammals (e.g., humans and mice) makes the chicken immune system recognize more epitopes on mammalian proteins. It generates a more diverse antibody repertoire [20–23]. Thus, the recombinant monoclonal antibody has many benefits, including higher sensitivity and specificity, lower cost, and suitability at the industrial scale.

Since no qualified chicken myeloma fusion-partner cell line is available for making chicken hybridomas, monoclonal IgYs can be selected with either antibody library strategies (such as phage display, ribosome display, or yeast display) or single B-cell cloning [24–27]. Herein, we immunized hens with 31-peptide of hTK1 to select monoclonal anti-TK1 IgY from a phage display immune scFv library and developed a novel hTK1-IgY-rmAb with high sensitivity and specificity that is stable. The detection of STK1p by hTK1-IgY-rmAb based on a new automatic chemiluminescence sandwich-BSA platform will replace the present detection system of hTK1-IgY-pAb together with the semiautomatic ECL dot blot assay for early tumour discovery in large-scale health screening.

## 2. Materials and Methods

### 2.1. Construction of Phage Display Hen scFv Library

#### 2.1.1. Hen Immunization and Total RNA Isolation

The immunizing antigen of the 31-peptide of hTK1 was synthesized and conjugated with KLH by Hybio Pharmaceutical, Shenzhen, China. Seven hens were immunized with the conjugates. One milligram of conjugates was resuspended in 500 *μ*L phosphate-buffered saline (PBS, pH 7.4) and mixed with 500 *μ*L complete Freund's adjuvant (Sigma-Aldrich, F5881) for initial immunization. The antigen was injected into the wings with multiple immunizations. A total of 0.5 mg of conjugates mixed with incomplete Freund's adjuvant (Sigma-Aldrich, F5506) was used for booster injections at 3, 5, and 9 weeks. After the fifth week of immunization, eggs were collected [28]. TK1-IgY-pAb was purified from egg yolk, and the specificity and sensitivity of TK1 by ELISA, Western blotting, IHC, and ECL dot blotting were evaluated as described by Wu et al. [28] and Skog et al. [9]. The immunizing antigen of the 31-peptide of hTK1 conjugated with BSA (Hybio Pharmaceutical, Shenzhen, China) was used as an hTK1 calibrator, and rhTK1 (from our laboratory) was used as a quality control. The hen was sacrificed to collect splenocytes that would be homogenized with TRIzol solution (Invitrogen, 15596026) and subjected to isolation of the total RNA for further RT-PCR.

#### 2.1.2. Library Construction

cDNA was prepared by RT-PCR using the PrimeScript™ 1st Strand cDNA Synthesis Kit (TaKaRa, 6110A). Then, the variable region of the heavy chain (VH) and the variable region of the light chain (VL) genes were amplified separately. The full-length scFv gene pool was made by an overlapping step. The primers are listed in Table 1. The PCR conditions were as follows: 94°C for 2 min and 25 cycles of 50°C for 30 s, 72°C for 30 s, and 94°C for 30 s.

**Figure 1:**
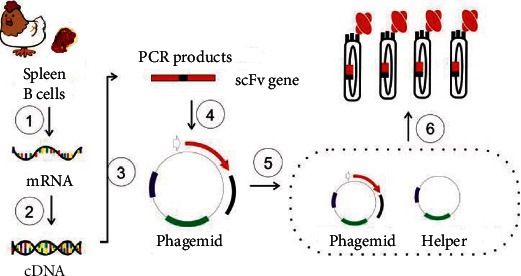
Schematic diagram of the construction of a large-capacity phage antibody display library: ①: mRNA isolation from hen B-cells; ②: RT-PCR; ③: scFv assembly by overlapping PCR; ④: ligation; ⑤: electrotransformation; ⑥: phage packaging.

The scFv gene pool was cloned into a phagemid (pSCD-1, Supplementary Figure 1) via SfiI/NotI with T4 DNA ligase. The ligation mixture was desalted and electrotransformed into TG1 bacterial-competent cells to make a bacterial pool. At OD_600_ = 1.0, M13KO7 helper phage was added to the culture of the bacterial pool to aid the packaging of the phage particles, which was the phage pool precipitated with PEG 6000 (Sigma-Aldrich, 81260) and titrated conventionally before being preserved at -80°C. The process is summarized in Figure 1.

#### 2.1.3. Affinity Panning

Altogether, three rounds of screening were performed by immobilizing either the TK1 calibrator or quality control with 96-well plates. The wells were blocked with PBSM (PBS-2% milk) before three washes with PBS. Then, the input phage pool was added to PBSM and incubated at 37°C for 1 hour. After 9 washes with PBST (0.1%-0.3% Tween-20 in PBS), the bound output phage pool was eluted by trypsin (Merck, T6567) digestion. The eluate was titrated by infecting TG1 bacterial cells and spreading them on ampicillin-containing LB plates. If a subsequent round of screening was needed, the eluate was amplified by infecting TG1 cells in a shaking flask and packaging them with the aid of M13KO7 helper phage (NEB, #N0315S). The amplified eluate was titrated conventionally as the input phage pool for the next round of screening.

If the enrichment factor (output/input of each round) increased, the enrichment effect appeared, and the final eluate was subjected to phage ELISA.

#### 2.1.4. Phage ELISA

The colonies on the titration plate of the final eluate were picked up and subjected to phage packaging in a 96-well plate. In brief, the colonies were seeded into 96-well plates in 100 *μ*L 2YT-AG (2YT medium, 100 *μ*g/mL ampicillin, 0.1% glucose). After overnight culturing, 20 *μ*L of culture per well was transferred into a new plate (packaging plate) with 80 *μ*L of fresh 2YT-A (2YT medium, 100 *μ*g/mL ampicillin) per well. When OD_600_ = 0.5, M13KO7 helper phage was added at an MOI (multiplicity of infection) = 20, followed by 180 rpm shaking at 37°C for 30 minutes. After that, 100 *μ*L 2YT-AK (2YT medium, 100 *μ*g/mL ampicillin, 50 *μ*g/mL kanamycin) was added to each well before shaking at 220 rpm at 37°C overnight. Finally, 100 *μ*L supernatant per well was transferred into a 96-well plate (the assay plate) precoated with hTK1 calibrator or quality control. The following step was completed routinely with HRP conjugate of rabbit anti-M13 polyclonal antibody as the secondary antibody and 3,3′,5,5′-tetramethylbenzidine (Merck, ES022) as the substrate. The positive clones were sequenced locally and analysed with Geneious Prime software (Biomatters, New Zealand).

### 2.2. Preparation of hTK1-IgY-rmAb

#### 2.2.1. Reformation of scFv into IgY

According to the limit concentration dilution method based on rhTK1 developed in our laboratory, we screened the scFv of positive clones and then reformatted them. We reformatted scFv into IgY by separate PCR amplification of the VH/VL gene and routine cloning into pTT5-CH/pTT5-CL (premade and derived from pTT5) between the IL2 signal peptide gene and hen constant region gene (CH1-CH2-CH3-CH4 gene/CL gene). The His tag was added at the C-terminus of the heavy chain to facilitate further purification with a Ni column/IMAC (immobilized metal affinity chromatograph). The expression vectors were pTT5-TK1-IgY-H (expressing heavy chain) and pTT5-TK1-IgY-L (expressing light chain).

#### 2.2.2. Expression and Purification of Recombinant TK1-IgY

Each clone's endotoxin-free plasmids pTT5-TK1-IgY-H and pTT5-TK1-IgY-L were isolated and cotransfected at a ratio of 1/3 into 293F cells (Invitrogen) with 25 kDa linear PEI (Polyplus-transfection, Merck, P3143). The cells were cultured with FreeStyle medium (QuaCell Biotechnology, A21501) in a shaking flask with a CO_2_ shaker. After 4 days of culture at 37°C, the supernatant was collected via centrifugation and loaded onto a Ni column. Then, the hTK1-IgY-rmAb was eluted with 200 mM imidazole in PBS after washing with 20 CV of PBS.

### 2.3. Characterization of the hTK1-IgY-rmAb

#### 2.3.1. Western Blotting

To confirm the specificity and sensitivity of hTK1-IgY-rmAb, native PAGE/Western blotting and ELISA were performed according to previous publications [28–30] with slight modifications. In brief, the TK1-positive cell line (human colon tumour TK1^+^: HT29) and TK1-negative cell line (human colon tumour TK^−^: 143B, TK1 gene knockout cells) were cultured to the logarithmic growth phase at a concentration of 1 × 10^7^ cells/mL. The cells were centrifuged (800 rpm for 8 min), and then, the cell pellet was resuspended in lysis buffer (50 mM Tris, 150 mM NaCl, and 1 mM EDTA, 1% NP40) at 4°C for 20 min. Then, the supernatant was collected via centrifugation at 15,000 rpm for 10 min. GAPDH (ORIGENE, TA802519s) was used as the control in the Western blotting experiments. Polyacrylamide gel electrophoresis (PAGE) was performed using a 4-10% gradient native polyacrylamide gel; GAPDH (1 : 1000) in 5 *μ*L of 143B TK^−^ and HT29 TK^+^ cell lysates was applied to the wells of the gel, followed by transfer to a protein-blotted polyvinylidene membrane (PVDF, Millipore, GS0914). The membrane was blocked with 10% skimmed milk for four hours, incubated with the hTK1-IgY-rmAb and anti-GAPDH mAb for one hour at room temperature, and then incubated with biotinylated donkey anti-chicken IgY secondary antibody (Jackson ImmunoResearch, 703-066-155) for one hour at room temperature. The protein bands were then detected using Immobilon Western chemiluminescent SA-HRP (streptavidin-HRP conjugate).

#### 2.3.2. ELISA

The hTK1-IgY-rmAb targeting the hTK1 calibrator was detected by ELISA following a previously reported method [28] with slight modifications. Briefly, 96-well plates (Thermo Fisher Scientific, 7905) were coated with 100 *μ*L hTK1 calibrators (31-peptide of hTK1 conjugate of BSA, 0.5 *μ*g/mL) per well in carbonate buffer (pH 9.6) and incubated overnight at 4°C. The wells were rinsed once, and blocking buffer was added (5% nonfat milk in PBS) for one hour at 37°C. The wells were rinsed, and then, the gel was incubated with purified hTK1-IgY-rmAb at different concentrations (7.8-1000 ng/mL) for one hour at 37°C, followed by incubation with biotinylated donkey anti-chicken IgY secondary antibody (Jackson ImmunoResearch, 703-066-155) for one hour at room temperature and then incubation with SA-HRP for one hour at room temperature. Then, the gel was incubated with tetramethylbenzidine (TMB; 100 *μ*L per well) at 37°C for 10 minutes. Then, 100 *μ*L of 1 M H_2_SO_4_ was added to each well to terminate the reaction. The plates were then scanned in a spectrophotometer at 450 nm.

#### 2.3.3. Affinity Binding, Sensitivity, and Specificity of the Purified hTK1-IgY-rmAbs

Purified hTK1-IgY-rmAbs are necessary to further evaluate affinity binding, sensitivity, and specificity. According to the SOP instructions of Sino-Swed Tong Kang Bio-Tech (http://www.sstkbiotech.com, China), for this assay, high-affinity binding: Kd (dissociation constant) < 10^−9^, high sensitivity: the slope of the linear curve declines less than 10% using hTK1 calibrators, and significant specificity: the *r* value is higher than 0.90 by the Pearson correlation test are required.

According to a previous study, a slightly modified limit dilution method was used to assess the affinity binding [31]. ELISA plates (Thermo Fisher Scientific, 7905) were coated with 100 *μ*L rhTK1 (0.5 *μ*g/mL) per well in carbonate buffer (pH 9.6) and incubated for 24 h at 4°C. The wells were washed with PBS-0.1% Tween 20 once, and 5% nonfat milk in PBS was used to block the wells for one hour at 37°C. Then, 5 *μ*g/mL hTK1-IgY-rmAb was added to the well at the starting concentration, and 2-fold gradient dilution was performed (16 gradients in total). After incubation for 1 h at 37°C, each well was washed three times with PBST and then incubated with biotinylated donkey anti-chicken IgY secondary antibody for one hour at 37°C. The assay wells were washed three times with 300 *μ*L PBST, followed by incubation with SA-HRP for one hour at 37°C. TMB (100 *μ*L/well) was added for 10 min, and the reaction was stopped with 100 *μ*L 1 M H_2_SO_4_. The plates were then scanned in a spectrophotometer at 450 nm. Finally, GraphPad Prism 8 was used to analyse the affinity binding data. An affinity binding of <10^−9^ Kd indicated high-affinity binding.

hTK1-IgY-pAb (“gold standard”) was used with an ECL dot blot assay kit according to the instructions of Sino-Swed Tong Kang Bio-Tech (http://www.sstkbiotech.com, China). The hTK1 calibrators were used for sensitivity detection. Standard serum samples supplied by Sino-Swed Tong Kang Bio-Tech were used for specificity detection. STK1p values in serum samples from voluntary blood donors (*n* = 18) were detected and divided into two groups: elevated-STK1p value (>2.0 pM, *n* = 10) and low-STK1p value (≤2.0 pM, *n* = 8) based on the risk threshold of STK1p = 2.0 pM for health screening [9]. All serum samples were supplied by Sino-Swed Tong Kang Bio-Tech (Shenzhen, China) and stored at -80°C. Briefly, three microlitres of the hTK1 calibrators corresponding to 2.2, 6.6, and 20 pM and 18 serum samples were also dotted on five nitrocellulose membranes (Millipore, Z358657). The samples were probed with five different hTK1-IgY-rmAbs. hTK1-IgY-pAb was used as a control in parallel (two repeated tests). The intensities of the spots on the membrane were determined by a CCD camera (CIS-l Imaging System, Sino-Swed Tong Kang Bio-Tech, Shenzhen, China). The values were calculated and expressed as OD and TK1 pM values according to the slope of the curve of the intensities of known hTK1 concentrations. According to our SOP document, (1) comparing the five hTK1-IgY-rmAbs and the hTK1-IgY-pAb using the hTK1 calibrators, the slope of the linear curve declined less than 10%, indicating that the sensitivity of hTK1-IgY-rmAb was sufficient, and (2) the Pearson correlation test was used to assess the specificity of the five different hTK1-IgY-rmAbs based on the immune response of serum TK1 when compared with that of hTK1-IgY-pAb. An *r* value higher than 0.90 indicates a highly significant correlation, indicating that hTK1-IgY-rmAb is sufficiently specific.

#### 2.3.4. IHC

Anti-mouse hTK1 mAb [9, 16] and anti-chicken hTK1 pAbs [28] were specific and sensitive for TK1 immunohistochemistry staining in patients with carcinoma. To confirm whether the hTK1-IgY-rmAb was specific and sensitive enough to recognize hTK1 in tumour cells of tissues, IHC was performed [9, 28] as reported previously with slight modification.

Three tissue specimens of tonsil inflammation patients who were histologically identified as having normal tonsils were collected. In the same patients, tissues from serous ovarian adenocarcinoma (grade 3) were also collected.

In brief, the tissue sections were dewaxed and hydrated, followed by treatment with an endogenous biotin-blocking kit (Invitrogen, E21390) to block endogenous biotin. The sections were incubated for 3 min in 3% H_2_O_2_ to block endogenous peroxidase. After incubation with hTK1-IgY-rmAb (2.5 *μ*g/mL, in PBS) overnight at 4°C, the sections were rinsed in PBS and incubated with biotinylated donkey anti-chicken IgY antibody (Jackson ImmunoResearch, 703-036-155) at room temperature for 60 min. After further washing with PBS 3 times, SA-HRP (Invitrogen, SA10001) was added, and the sections were incubated at room temperature for 90 min. Fresh diaminobenzidine (DAB) solution was used for colour rendering, and the slides were slightly counterstained with haematoxylin. The numbers of TK1-stained tumour and normal cells were counted among 100 cells at 400x magnification and classified into four groups: ≤5%: “-”; 6-25%: “+”; 26-50%: “++”; and ≥50%: “+++.” The percentages of labelled TK1 cells were denoted as the labelling index (LI). An LI index above 5% was denoted as “positive LI.” In most cases, TK1 in stained normal cells was less than 5%.

#### 2.3.5. Serum Samples

A total of 292 blood samples donated voluntarily at 3 different health centres (Changzhou Cancer Hospital (*n* = 50), Shenzhen Charity Hospital (*n* = 83), and Longhua Community Hospitals for a livelihood project authorized by the Bureau of Civil Affairs of Shenzhen City (*n* = 159)) were included in this study.

The 292 voluntary blood donors included 83 men and 209 women, and their mean age was 38.9 years (19-72 years). No malignant tumours or infectious diseases were self-reported. Serum samples were collected from July to December 2021 and stored at -80°C until analysis.

#### 2.3.6. STK1p Assay

**Figure 2:**
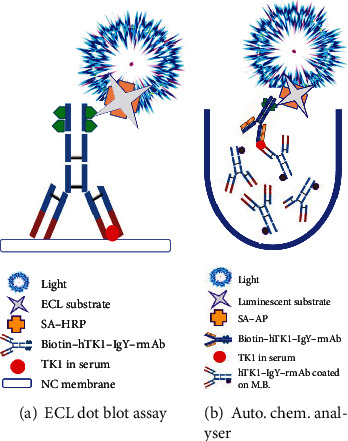
Schematic diagram of the two-assay system using hTK1-IgY-rmAb. The detection of STK1p using hTK1-IgY-rmAb on the semiautomatic ECL dot blot assay with BSA platform (a) and the novel automatic chemiluminescence analyser with sandwich-BSA platform (b). M.B.: magnetic bead.

TK1 in serum is rather low or undetectable in disease-free healthy persons (0.01-2.0 pM) [9]. A highly sensitive detection system is needed. We set up a semiautomatic ECL dot blot assay with the BSA platform for STK1p detection, which is reliable for detecting STK1 at a concentration of 0.01 pM [9]. Compared to the ECL assay without BSAS (0.2-0.3 pM), the sensitivity was improved by 20-30-fold (data not shown). To confirm the replacement of the TK1-IgY-pAb with the hTK1-IgY-rmAb for early tumour discovery, two types of STK1p assays were used (Figures 2(a) and 2(b)): (1) a semiautomatic ECL dot blot assay with the BSAS platform [9] and (2) an automatic chemiluminescence analyser (Keysmile, 6500) with the sandwich-BSA platform developed by us [9]. The lowest detectable concentration was 0.01 pM.

#### 2.3.7. Detection of STK1p by an Automatic Chemiluminescence Analyser with the BSAS Platform

First, the magnetic beads were activated according to the protocols of MS300 Tosyl magnetic beads (JSR Life Sciences J-MS-S300T). Briefly, 5 mg of MS300 Tosyl magnetic beads at a volume of 50 *μ*L was suspended in a centrifuge tube; then, the tube was placed on a magnetic field for 1 min, and the supernatant was removed. Next, 10 times activation buffer was used to shock wash the magnetic beads three times. After that, the supernatant was removed. The hTK1-IgY-rmAb was added to the magnetic beads at a ratio of 100 : 1, and 1/10 of the total volume of the catalytic reagent solution was added. The solutions were mixed, and then, the beads were rotated for 18 hours at 37°C. The coupling reaction was blocked by adding 10% BSA and allowing the reaction to continue for 6 hours at 37°C. The supernatant was removed by placing the mixture in a magnetic field, washing the magnetic TK1 beads with a cleaning solution three times, diluting to 1 mg/mL, and storing at 2-8°C.

Second, the hTK1-IgY-rmAb was diluted to 1 mg/mL and biotinylated using an EZ-Link™ Sulfo-NHS-LC-Biotinylation Kit (Thermo Scientific™, 21435) according to the manufacturer's instructions. Later, the excess biotin reagent was removed using a desalting column and stored at 4°C.

Third, 10 *μ*L of serum samples or hTK1 calibrators were loaded into the analyser according to the operation manual (KeySmart Ltd., China). The samples and 30 *μ*L of hTK1-IgY-rmAb antibody-coated magnetic beads were incubated at 37°C for 10 min to form a complex, and then, 10 *μ*L of biotinylated hTK1-IgY-rmAb antibody was added to form a double antibody sandwich complex with the complex formed in the first step and washed 3 times. Then, 100 *μ*L SA-HRP was added for 10 minutes at 37°C, and the beads were washed 3 times.

Finally, the chemiluminescence substrate was injected into the reaction tube after magnetic separation, the tube was incubated for 5 min, and the luminescence value was read. The content of STK1p in the sample was positively correlated with the luminescence value, and the content of STK1p was calculated from the luminescence standard curve.

### 2.4. Statistical Analysis

The statistical significance for the correlations between parameters was calculated by the Pearson correlation test (SPSS Statistics V25.0, IBM, USA). An *r* value > 0.75 was considered a qualified correlation, and an *r* value > 0.90 indicated a significant correlation.

## 3. Results

### 3.1. Construction of a Phage Display Hen scFv Library

**Figure 3:**
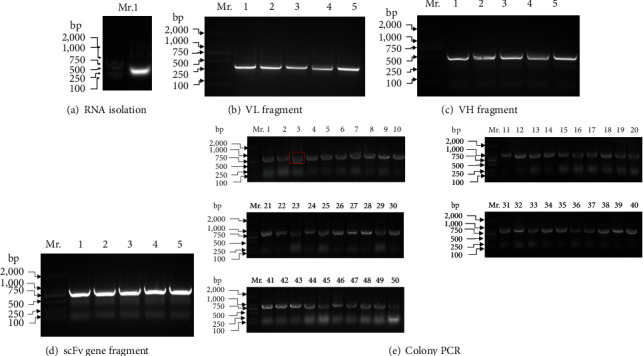
Summary of the construction of hTK1-IgY-scFv phage antibody libraries from immunized hens: (a) agarose gel electrophoresis detected the isolated RNA; (b) the antibody VL fragment was obtained by RT-PCR; (c) the VH fragment of the antibody was obtained by RT-PCR; (d) the scFv gene fragment was obtained by overlap PCR; (e) colony PCR was performed on 50 random clones in a total of 340 clones. A negative clone was detected (#3 in red box). Lane Mr. DNA marker: DNA 2000, 1000, 750, 500, 250, and 100 bp.

As shown in Figure 3, we isolated total RNA from TRIzol lysates of spleen cells. One band was detected corresponding to 28S (RNA sedimentation coefficient, Figure 3(a)). Then, reverse transcription was conducted to make a single chain of cDNA (data not shown). With cDNA as the template, VL (Figure 3(b)) or VH gene fragments (Figure 3(c)) were amplified by PCR using gene-specific primers of approximately 300 bp. Thus, the scFv gene (linking VL and VH with a flexible peptide, GGGGSGGGGSGGGGS) was made by overlapping PCR and was approximately 750 bp, as shown in Figure 3(d). The scFv fragments were inserted into the phagemid, and the positive insertion rate was tested by colony PCR. Ninety-eight percent of the clones in the library were predicted to carry scFv gene insertions, as shown in Figure 3(e). The primers were located in the region directly upstream and downstream of the scFv gene. Therefore, the PCR products are larger than the scFv genes. As the band in Lane 3 was much smaller than those from the other lanes, it was considered a negative clone. Finally, two primers (L1: TGGAATTGTGAGCGGATAACAATT and Myc-d: ATTCAGATCCTCTTCTGAGAT) were used for colony PCR. We produced a phage display hen scFv library of 3.4 × 10^9^ diversity.

### 3.2. Affinity Panning and Phage ELISA

The library was panned against an immobilized BSA conjugate of the 31-peptide of hTK1 by directly coating in 96-well plates. As a result, the enrichment effect was very strong after two rounds of screening, and all clones picked up from the second eluate, and then, the rhTK1 was directly coated in 96-well plates to perform the third panning. The ratios of output and input phage increased steadily after each round, with an approximately 920-fold increase in phage recovery after the third round compared with that after the first round of selection, demonstrating an efficient enrichment of specific antibodies, as shown in Table 2. Almost 800 clones were picked from the titration plate of the second and third eluates separately, and 13 positive clones were finally identified by phage ELISA, recognizing the 31-peptide of hTK1 (Supplementary tables). By local DNA sequencing, five unique scFvs were identified.

### 3.3. Characterization of the Purified hTK1-IgY-rmAbs

The five hTK1-IgY-rmAbs reformatted from five unique sequences of scFvs were named hTK1-IgY-rmAb#1, #2, #3, #4, and #5. They were transiently expressed by cotransfection of 293F cells with pTT5-TK1-IgY-H and pTT5-TK1-IgY-L of each hTK1-IgY-rmAb. The hTK1-IgY-rmAbs were routinely purified with a Ni column.

**Figure 4:**
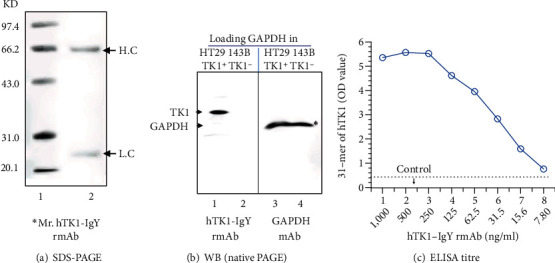
Specificity and sensitivity of hTK1-IgY-rmAb#5: (a) SDS-PAGE detection of the purified antibody (0.5 *μ*g). The IgY molecule heavy chain (H) was ≈66 kDa, and the light chain (L) was ≈25 kDa. (b) Western blot detection of antibody specificity; TK1-negative cell lines (143BTK1^−^) and TK1-positive cell lines (HT29) were used, and GAPDH was used as a loading control. (c) ELISA titre detected the #5 antibody. ^∗^Mr.: molecular weight marker.

**Figure 5:**
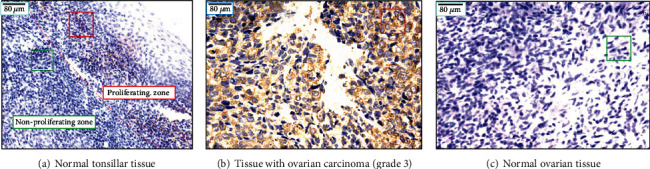
TK1 IHC staining of hTK1-IgY-rmAb#5: (a) TK1 staining of normal tonsil tissue; (b) a patient with ovarian serous adenocarcinoma (grade 3). TK1 staining is observed in brownish-yellow, mainly in the cytoplasm (examples of TK1 in the red box). (c) Normal ovarian tissue was taken from the far outside of the total ovarian serous adenocarcinoma specimen as a standard control (normal spindle cells are shown in green box). Blue staining was used to counterstain nuclei with haematoxylin. Magnification 200x.

**Figure 6:**
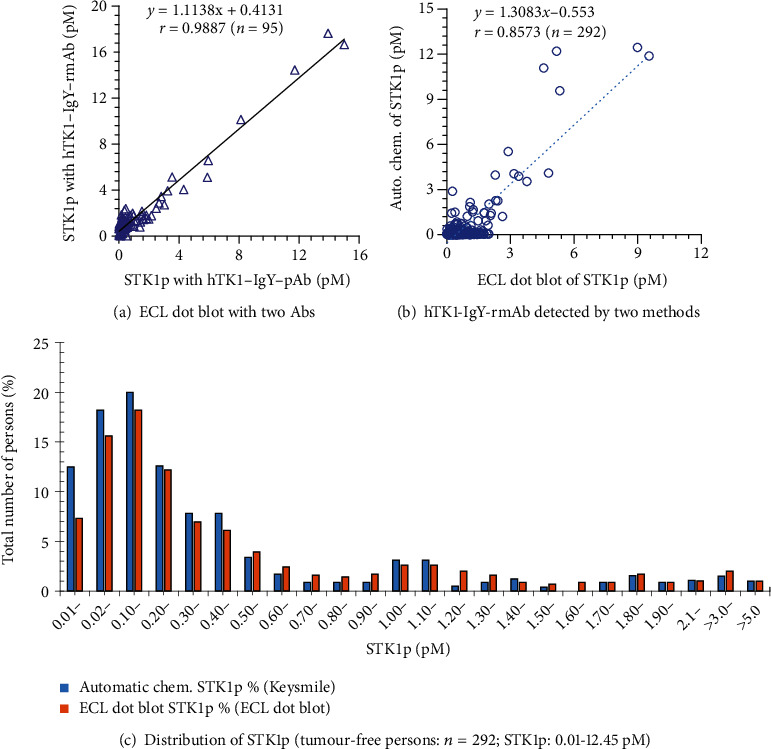
STK1p assay: (a) comparative study of the semiautomatic ECL dot blot assay between hTK1-IgY-pAb and hTK1-IgY-rmAb#5. Number of serum samples = 95 (STK1p, pM). (b) Comparative study of the semiautomatic ECL dot blot assay with the BSA platform and the automatic chemiluminescence analyser with the sandwich-BSA platform using hTK1-IgY-rmAb#5 (four batches). Number of serum samples = 292 (STK1p, pM). (c) Distribution of the concentration of STK1p (pM). ^∗^*r*: Pearson correlation test.

An optimal mAb must have high-affinity binding and must be sensitive and specific to recognize both calibrators of hTK1 and quality controls of rhTK1. The comparative determination of the affinity binding, sensitivity, and specificity between hTK1-IgY-pAb and the five different rmAbs is shown in Tables 3(a) and 3(b). The results demonstrated that compared to hTK1-IgY-pAb, only hTK1-IgY-rmAb#5 showed high-affinity binding (3.95 × 10^−10^), high sensitivity (slope 89.98), and high specificity (*r* ≈ 0.92-0.963). The rmAbs #1, #2, #3, and #4 failed to do that. Thus, we chose only hTK1-IgY-rmAb#5 and continued to determine the characteristics of the rmAb (Figures 4–6 and Table 4).

### 3.4. Confirmation of the Binding of hTK1-IgY-rmAb#5 to Native TK1 by ELISA, Western Blot, and IHC

According to the results of reduced SDS-PAGE, the heavy chain (H) was ≈66 kDa, and the light chain (L) was ≈25 kDa (Figure 4(a)), which was similar to the results of a previous study. The total molecular weight of the TK1-IgY Ab was ≈180 kDa [32].

The binding of hTK1-IgY-rmAb#5 to native TK1 in the lysate was verified by native Western blotting. A specific band in the TK1^+^ cell line (HT29) was observed, but no TK1 band in the TK1^−^negative cell line (143B) was observed (Figure 4(b)). Only the GAPDH band was observed when the GAPDH mAb was used for probing in the loading GAPDH as the control (Figure 4(b)). Thus, hTK1-IgY-rmAb#5 only recognized TK1 in the positive cell line (HT29 TK1^+^).

The results of TK1 IHC staining of hTK1-IgY-rmAb#5 are shown in Figure 5(a) (normal tonsil tissue), Figure 5(b) (ovarian cancer tissue, grade 3), and Figure 5(c) (ovarian normal tissue). The normal tissue is taken from a distant part of the tumour tissue and contains nonproliferating or proliferating cells. Pathological staining (haematoxylin) can identify the area of normal and malignant tissue but not the rate of cell proliferation. It is well known that TK1 is a reliable cell proliferation marker. Thus, TK1 IHC determines the number and intensity of stained cells showing the cell proliferation rate, including normal cells and tumour cells, which is important to know when trying to assess a true cell proliferation rate.

Normal tonsil tissue contains two types of zones, a proliferation zone and a nonproliferating zone; therefore, the tonsil can be used as a standard control [9] when assessing the specificity and sensitivity of our hTK1-IgY-rmAb#5. IHC for TK1 showed brown-yellow staining only in the cytoplasm of the proliferating zone (score > 25%) and not in the nonproliferating zone (score < 5%), confirming that hTK1-IgY-rmAb#5 is highly specific and sensitive. Furthermore, strong staining of TK1 in the cytoplasm (score > 50%) was also observed in the area with poorly differentiated ovarian cancer tumour tissue (grade 3), but little staining was observed in tissue far distant from the ovarian tumour tissue (score < 5%), confirming that hTK1-IgY-rmAb#5 is highly specific and sufficiently sensitive. It can be used as an optimal serum biomarker in health screening and clinical oncology settings. Here, an example photo shows TK1 IHC staining of hTK1-Ig-rmAb#5 in normal tonsil tissue (Figure 5(a)), ovarian cancer tissue (Figure 5(b)), and normal ovarian tissue (Figure 5(c)).

### 3.5. Serological Assay with hTK1-IgY-rmAb#5

STK1p was detected in 95 voluntary blood donors (no malignant tumours or infectious diseases were self-reported) using either TK1-IgY-pAb or hTK1-IgY-rmAb#5 based on the semiautomatic ECL dot blot assay. As shown in Figure 6(a), the coincidence rate of the Pearson test showed *r* = 0.988, confirming that the replacement of TK1-IgY-pAb with hTK1-IgY-rmAb#5 is reliable.

To confirm the stability of hTK1-IgY-rmAb#5, a comparative study between an automatic chemiluminescence analyser and the ECL dot blot assay was performed using 4 different batches of hTK1-IgY-rmAb#5. The STK1p results of 292 serum samples from three different health management groups (no malignant tumours or infectious diseases were self-reported) are summarized in Table 4 and Figure 6(b). The coincidence rate (Pearson correlation test) between the two methods of different batches of hTK1-IgY-rmAb#5 is presented in Table 4. The coincidence rate in 292 serum samples was *r* = 0.857 (Figure 6(b)), exceeding *r* = 0.75, which implied that the two detection methods have a good coincidence rate. HTK1-IgY-rmAb#5 is highly stable, and the automatic chemiluminescence analyser can replace the ECL dot blot assay. The number of people with elevated STK1p values (>2 pM) in the 292 voluntary blood donors was similar when using the ECL dot blot assay (4.8% (14/292)) or the automatic chemiluminescence analyser (4.5% (13/292)) (Figure 6(b)).

The lowest detectable value of STK1p on the semiautomatic ECL dot blot assay BSA platform and the automatic chemiluminescence analyser with the sandwich-BSA platform was 0.01 pM, in agreement with the recently published paper based on the IgY poly-antibody against STK1p by the same automatic chemiluminescence platform [33]. The number of patients with 0.01 pM was approximately 8-10% of the total tumour population. Therefore, the highly stable hTK1-IgY-rmAb#5 can not only replace hTK1-IgY-pAb in the present commercial dot blot assay but also be used in developing a novel automatic chemiluminescence analyser with the sandwich-BSA platform.

## 4. Discussion

Cancer is a chronic disease of abnormal regulation of cell growth. More specifically, cancer is a molecular genetic disease [34]. It is a manifestation of dysfunctional regulation of normal genomic processes responsible for cell differentiation and unlimited cell proliferation over a 10- to 30-year period. TK1 is a reliable tumour proliferation and prognostic biomarker [9] that can be used for all types of tumours. In the past 30 years, we have investigated the use of STK1p as a specific prognostic biomarker for predicting the tumour growth rate in cancer patients and for predicting the risk of tumour progression. We successfully developed a serum test based on chicken TK1-IgY-pAb against the C-terminal peptide 195-225 of human TK1 [9].

The benefit of STK1p is that it increases in serum before imaging detection, which is useful for detecting the risk of tumour progression. An investigation based on real-world data of 35,365 participants at four independent health screening centres showed that the AUC value (area under the curve in the ROC analysis) of STK1p was 0.96, the likelihood (+) value was 236.5, and the sensitivity and specificity were 0.78 and 0.997, respectively. With 132 months of follow-up, randomly selected people with elevated STK1p values (170/702) showed a four times higher risk of developing malignancies than people with low STK1p values (*n* = 6,352/26,484) [35]. Based on these promising results, a commercial ECL dot blot test was developed to detect the concentration of STK1p, replacing the STKa detection method [9]. We suggest that people continue regular health examinations, particularly if they have persistently high STK1p values. It is important to give people the best opportunity for the early treatment and cure of tumours [36].

In the past few years, monoclonal IgG antibodies have mainly been used in clinical diagnosis. IgG is the main antibody type produced by the immune system of mammals and exists in blood and tissue fluid, which has a high affinity for antigens [37]. However, due to the RF and HAMA reaction, it potentially gives false-positive results. IgY is highly homologous to mammalian IgG antibody. Because of the lack of a hinge region compared to the IgG structure, IgY does not react with certain components of the human immune system and shows greater affinity for mammalian conserved proteins. Therefore, IgY is more suitable for diagnostic purposes than mammalian antibodies [21].

Chicken IgY is usually produced by the immunization of hens and purification from egg yolks [21]. The 31-peptide of hTK1 (C-terminus, residues 195-225) was designed to immunize hens to produce TK1-IgY-pAb. A large amount of TK1-IgY-pAb can be purified from egg yolk with 31-peptide of hTK1-based affinity chromatography [28]. However, due to the individual differences among the hens being immunized, the quality of TK1-IgY-pAb is difficult to control even when a SOP designed by the manufacturer is executed. The TK1-IgY-pAb from each hen must be carefully examined, limiting the wider application of the test. Compared with traditional IgY polyclonal antibodies, the production of recombinant monoclonal antibodies requires fewer animals (animals are only needed before the antibody library is established), and the antibodies produced by stable CHO cell lines have a short production cycle and small batch difference. There are commercially available recombinant TK1 antibodies. However, none are useful as serological biomarkers in health screening or clinical oncology.

Herein, we decided to develop a chicken-based hTK1-IgY-rmAb by constructing and screening a phage display immune scFv library, overcoming the above disadvantages of TK1-IgY-pAb, and helping in the development of more novel tests in the future. HTK1-IgY-rmAb should recognize the common epitope among the TK1-derived peptide, rhTK1, and STK1 for the STK1p assay.

We immunized hens with the 31-peptide of hTK1. The chemically synthesized 31-peptide contains at least 5 specific nonnative epitopes beyond full-length TK1 [9]. The epitopes are more immunogenic to evoke B-cell clones to produce unwanted IgY antibodies. When panning against the 31-peptide of hTK1, all tested clones only bound to the 31-peptide of hTK1. The rare B-cell clones should produce IgY recognizing the native common epitopes. Accordingly, we panned against immobilizing full-length TK1 and successfully captured the rare clones binding to both the 31-peptide of hTK1 and rhTK1.

Affinity panning is an enriching process that allows the capture of rare clones within a short time [38, 39]. When adopting a single B-cell cloning strategy, many more clones have to be tested in parallel to identify rare ones. Full-length TK1 is not stable enough for labelling with biotin or fluorescent dye, so FACS of rare B-cell clones is impossible [40]. This is why we adopted the phage display antibody library strategy here.

In this study, we identified five reformatted hTK1-IgY-rmAbs. The results showed that only hTK1-IgY-rmAb#5 had high-affinity binding (3.95 × 10^−10^ mol/L) with rhTK1 (quality control), high sensitivity with hTK1 calibrators (slope of linear curve = 89.98), and highly significant correlation to low/elevated STK1p (*r* ≈ 0.92-0.963) (Table 3). Thus, hTK1-IgY-rmAb#5 is the sole triple-binding hTK1-IgY-rmAb. Western blotting with TK1-positive/negative cell lysates and IHC of normal tonsil tissue and patients with ovarian serous adenocarcinoma tissue further confirmed the binding of hTK1-IgY-rmAb#5 to native TK1. hTK1-IgY-rmAb#5 has the potential to be developed as a clinical IHC antibody. Furthermore, the TK1 serum samples from health centres showed high coincidence rates (*r* = 0.988, *n* = 90) between hTK1-IgY-rmAb#5 and hTK1-IgY-pAb and between the semiautomatic ECL dot blot BSA platform and the novel automatic chemiluminescence sandwich-BSA platform (*r* = 0.857, *n* = 292, Figure 6). hTK1-IgY-rmAb#5 is stable and highly sensitive, detecting STK1p at a concentration of 0.01 pM. The accuracy is high (SD < 2.5%) between different batches (Table 4).

The IgY-pAbs are purified from egg yolk, and the egg comes from hens immune to specific antigens. Then, high-titre-specific antibodies were purified. The whole production cycle is long. However, approximately 0.6-15 mg of antigen-specific antibodies can be produced, and the yield is low [24]. However, IgY-rmAb does not need animal immunization and thus greatly shortens the production cycle; it has a high yield, low production cost, high specificity, etc. In addition, IgY-rmAb can be expressed by building a stable CHO cell line; its yield can be up to 5 g/L, and the differences in antibody batches between different batches are small [41, 42].

In the past few years, we detected STK1p by a dot blot BSA platform. This traditional detection method has less sample demand, low cost, and high specificity. However, the whole operation process is complicated, time-consuming, and easily affected by the environment and has high technical requirements for operators. Therefore, more rapid and efficient detection techniques are needed. In general, IgY-rmAbs can solve the problem we encountered. The automatic chemiluminescence analyser with a sandwich-BSA platform improved the sensitivity by adding biotin. Thus, the automatic chemiluminescence sandwich-BSA platform is a technique with higher accuracy than the dot blot assay. This method is beneficial when running a larger sample size.

The combination of IgY-rmAb and the new analyser will be extremely useful when developing new serum biomarkers for early tumour discovery in large-scale health screenings.

The novel hTK1-IgY-rmAb assay on a new automatic chemiluminescence sandwich-BSA platform is easy to perform and beneficial for large-scale health screenings.

## 5. Conclusions

With the hTK1-derived 31-peptide as an immunogen, a phage display immune library was constructed and screened to select an hTK1-IgY-rmAb (#5). The hTK1-IgY-rmAb has a stable, high-affinity immune response with 31-peptide of hTK1, rTK1, and TK1 in serum based on a new automatic chemiluminescence sandwich-BSA platform. The hTK1-IgY-rmAb combined with an automatic chemiluminescence sandwich-BSA platform can provide more accurate and stable detection of STK1p data. The detection of STK1p by hTK1-IgY-rmAb based on the new automatic chemiluminescence sandwich-BSA platform will replace the present detection system of hTK1-IgY-pAb on a semiautomatic ECL dot blot assay for early tumour discovery in large-scale health screening in the future.

## Figures and Tables

**Table 1:** The primers used in the construction of the phage display hen scFv library.

Primers	Sequences (5′-3′)	PCR steps
VH-B1	GTCATCAGGCCCAGCCGGCCATGGCCGCCGTGACGTTGGACGAGTCCGG	VH-1^st^
VH-F1	GCCTGAGCCGCCACCACCGGAGGAGACGATGACTTCGGT	
VL-B1-1	AGTGGTGGAGGAGGATCCCAGGCAGCGCTGACTCAGCC	VL-1^st^
VL-B1-2	AGTGGTGGAGGAGGATCCCAGGCAGCACTGACTCAGCC	
VL-B1-3	AGTGGTGGAGGAGGATCCCAGGCAGCGCTGACTCTGCC	
VL-B1-4	AGTGGTGGAGGAGGATCCCAGGCAGCAGTGACTCAGCC	
VL-B1-5	AGTGGTGGAGGAGGATCCCAGGCAGCACCGACTCAGCT	
VL-F1	GTTACTGGTTTGCCTGCGGCCGCTAGGACGGTCAGGGTTGTCCC	
VH-B2	CTTAGAGTCATCAGGCCCAGCCGGCCATGGCC	VH-2^nd^
VH-F2	TCCTCCACCACTTCCGCCGCCGCCTGAGCCGCCACCACC	
VL-B2	CGGCTCAGGCGGCGGCGGAAGTGGTGGAGGAGGATCC	VL-2^nd^
VL-F2	CGATGGTTAGCGTTACTGGTTTGCCTGC	
VH-B3	CTTAGAGTCATCAGGCCCAGCCGGCC	scFv-3^rd^
VL-F3	CGATGGTTAGCGTTACTGGTTTG	

**Table 2:** Phages were applied and eluted in each round of panning by phage display.

Round	Input (pfu^∗^)	Output (pfu^∗^)	Ratio (%)
1	5.0 × 10^12^	9.6 × 10^3^	1.92 × 10^−9^
2	5.24 × 10^12^	7.44 × 10^4^	1.42 × 10^−8^
3	5.28 × 10^12^	9.36 × 10^6^	1.77 × 10^−6^

^∗^pfu: plaque-forming unit.

**Table:** (a) Comparison of sensitivity between hTK1-IgY-pAb (pAb) and the five hTK1-IgY-rmAbs (rmAb)

Type of IgY Ab	Affinity (rhTK1)^∗^		Sensitivity (calibrators)^∗∗^	
	Kd value	Q^‡^, <10^−9^ Kd	Slope	Q^‡^, <10%
pAb vs.	4.282 × 10^−11^	Yes	89.98	
rmAb #1	4.70 × 10^−8^	Not	89.94	(0.04%) yes
rmAb #2	2.07 × 10^−10^	Not	83.51	(7.20%) yes
rmAb #3	1.91 × 10^−8^	Not	89.56	(0.46%) yes
rmAb #4	2.57 × 10^−9^	Yes	89.12	(0.95%) yes
rmAb #5	3.95 × 10^−10^	Yes	89.98	(0.00%) yes

**Table:** (b) Comparison of specificity between hTK1-IgY-pAb (pAb) and the five hTK1-IgY rmAbs (rmAb) (TK1 in serum)

pAb vs. rmAb	*r* value at low STK1p	Q^‡^, *r*^∗∗∗^ > 0.90	*r* value at elevated STK1p	Q^‡^, *r*^∗∗∗^ > 0.90
rmAb #1	0.467	Not	0.512	Not
rmAb #2	0.181	Not	0.457	Not
rmAb #3	0.105	Not	0.409	Not
rmAb #4	0.964	Yes	0.890	Not
rmAb #5	0.920	Yes	0.963	Yes

^∗^The affinity binder had a Kd value < 10^−9^ when high-affinity binding was achieved; ^∗∗^sensitivity: when the slope (linearity of the calibrator curve) using the hTK1-IgY-rmAb declined less than 10%, it indicated that the rmAb was sufficiently sensitive; ^∗∗∗^*r*: *r* value was tested by Pearson correlation. An *r* value > 0.9 was considered a highly significant correlation. Kd: dissociation constant. ^‡^Q: qualified.

**Table:** (a) STK1p assay. The correlation score was analysed in batches of hTK1-IgY-rmAb# 5 using the semiautomatic ECL dot blot assay with the BSA platform

Source of serum samples	*r* ^∗^ value	Display range^∗∗^
	Pearson correlation	Different batch (OD)
Changzhou Cancer Hospital (*n* = 50)	0.811	0-1502
Shenzhen Charity Hospital (*n* = 83)	0.848	0-1438
Shenzhen Longhua Com. Hospital (W13, *n* = 80)	0.825	0-1722
Shenzhen Longhua Com. Hospital (W18, *n* = 79)	0.953	0-1638
Total (*n* = 292)	0.875	

Shenzhen Longhua Community Hospital W13 and 18. ^∗^*r*: Pearson correlation score; ^∗∗^display range indicates that the data are reliable in this range.

**Table:** (b) HTK1 calibrators were used to evaluate the stability and accuracy in four batches of hTK1-IgY-rmAb#5 using the semiautomatic ECL dot blot assay with the BSA platform and the automatic chemiluminescence analyser with the sandwich-BSA platform. Pearson correlation score (*r* > 99.9, data not shown)

hTK1 calibrator (pM)	Mean ± SD (OD value)	CV%
ECL dot blot		
2.2	11,029 ± 860.2	7.80
6.6	26,636 ± 2,087.9	7.92
20	99,985 ± 4,950.3	4.95
Automatic chemiluminescence		
1	753,128 ± 18,828.2	2.50
2	1,481,435 ± 16,295.8	1.10
5	3,638,642 ± 40,025.1	1.10
10	7,420,457 ± 51,943.2	0.70
20	14,854,175 ± 65,358.4	0.44

## Data Availability

The labelled dataset used to support the findings of this study is available from the corresponding authors upon request.

## Supplementary Materials

Supplementary Materials Supplementary Figure 1 shows the schematic map of phagemid pSCD-1. Supplementary tables were the results of phage ELISA.
