# Supplementary material for: Development of a Novel Recombinant Full-Length IgY Monoclonal Antibody against Human Thymidine Kinase 1 for Automatic Chemiluminescence Analysis on a Sandwich Biotin-Streptavidin Platform for Early Tumour Discovery
Source: J Immunol Res. 2023 Mar 17;2023:7612566. doi: 10.1155/2023/7612566 (PMC10038734; doi:10.1155/2023/7612566)

- Supplementary Figure1:


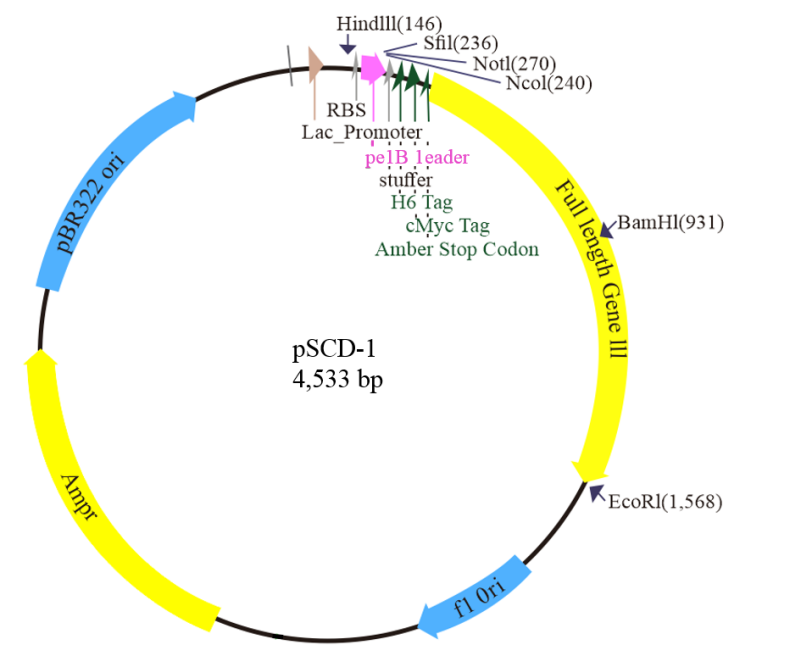


**Figure 1**. Schematic map of phagemid pSCD-1.

- Supplementary tables: **The results of phage ELISA;** The 13 dual-positive clones identified by phage ELISA, recognizing the 31-peptide of hTK1 are highlighted in red

**Negative control:** target molecule was coated, and phage M13KO7 was added as control

**Blank control:** coated with target molecules, and 1%M-PBS was added instead of phage

**Positive control:** directly coated with M13KO7

**Secondary antibody:** HRP conjugate of rabbit anti-M13 polyclonal antibody (Merck, ES022)

**31AA-BSA**: 31-peptide of hTK1 conjugate of BSA

**Control:** without coated antigen

| Clone number | 0.5ug/ml 31AA-BSA | Control |
| --- | --- | --- |
| 1 | 0.05 | 0.063 |
| 2 | 0.054 | 0.069 |
| 3 | 0.054 | 0.069 |
| 4 | 0.059 | 0.073 |
| 5 | 0.056 | 0.072 |
| 6 | 0.056 | 0.07 |
| 7 | 0.053 | 0.057 |
| 8 | 0.051 | 0.059 |
| 9 | 0.049 | 0.076 |
| 10 | 0.05 | 0.068 |
| 11 | 0.049 | 0.078 |
| 12 | 0.05 | 0.076 |
| 13 | 0.05 | 0.078 |
| 14 | 0.047 | 0.073 |
| 15 | 0.047 | 0.067 |
| 16 | 0.048 | 0.058 |
| 17 | 0.051 | 0.057 |
| 18 | 0.115 | 0.068 |
| 19 | 0.048 | 0.081 |
| 20 | 0.085 | 0.084 |
| 21 | 0.05 | 0.076 |
| 22 | 0.048 | 0.074 |
| 23 | 0.05 | 0.061 |
| 24 | 0.047 | 0.053 |
| 25 | 0.052 | 0.056 |
| 26 | 0.048 | 0.067 |
| 27 | 0.049 | 0.08 |
| 28 | 0.049 | 0.082 |
| 29 | 0.052 | 0.077 |
| 30 | 0.05 | 0.072 |
| 31 | 0.049 | 0.069 |
| 32 | 0.048 | 0.056 |
| 33 | 0.05 | 0.062 |
| 34 | 0.078 | 0.062 |
| 35 | 0.048 | 0.074 |
| 36 | 0.048 | 0.089 |
| 37 | 0.047 | 0.078 |
| 38 | 0.047 | 0.074 |
| 39 | 0.071 | 0.067 |
| 40 | 0.051 | 0.057 |
| 41 | 0.051 | 0.095 |
| 42 | 0.308 | 0.093 |
| 43 | 0.05 | 0.095 |
| 44 | 0.048 | 0.103 |
| 45 | 0.05 | 0.102 |
| 46 | 0.047 | 0.091 |
| 47 | 0.049 | 0.094 |
| 48 | 0.074 | 0.106 |
| 49 | 0.099 | 0.055 |
| 50 | 0.047 | 0.064 |
| 51 | 0.049 | 0.072 |
| 52 | 0.048 | 0.074 |
| 53 | 0.048 | 0.083 |
| 54 | 0.087 | 0.074 |
| 55 | 0.05 | 0.07 |
| 56 | 0.05 | 0.057 |
| 57 | 0.115 | 0.054 |
| 58 | 0.098 | 0.062 |
| 59 | 0.048 | 0.062 |
| 60 | 0.048 | 0.079 |
| 61 | 0.048 | 0.073 |
| 62 | 0.047 | 0.069 |
| 63 | 0.049 | 0.066 |
| 64 | 0.048 | 0.064 |
| 65 | 0.05 | 0.055 |
| 66 | 0.356 | 0.058 |
| 67 | 0.048 | 0.063 |
| 68 | 0.047 | 0.074 |
| 69 | 0.123 | 0.069 |
| 70 | 0.046 | 0.069 |
| 71 | 0.261 | 0.065 |
| 72 | 0.048 | 0.056 |
| 73 | 0.113 | 0.056 |
| 74 | 0.079 | 0.056 |
| 75 | 0.05 | 0.058 |
| 76 | 0.082 | 0.067 |
| 77 | 0.049 | 0.065 |
| 78 | 0.047 | 0.065 |
| 79 | 0.048 | 0.062 |
| 80 | 0.05 | 0.062 |
| 81 | 0.049 | 0.057 |
| 82 | 2.335 | 0.055 |
| 83 | 0.048 | 0.064 |
| 84 | 0.047 | 0.064 |
| 85 | 0.086 | 0.061 |
| 86 | 0.088 | 0.056 |
| 87 | 0.049 | 0.058 |
| 88 | 0.122 | 0.058 |
| 89 | 0.106 | 0.052 |
| 90 | 0.049 | 0.075 |
| 91 | 0.049 | 0.052 |
| 92 | 0.047 | 0.054 |
| 93 | 0.052 | 0.058 |
| Negative Control | 0.051 | 0.05 |
| Blank Control | 0.048 | 0.051 |
| Positive Control：1.2680 | | |


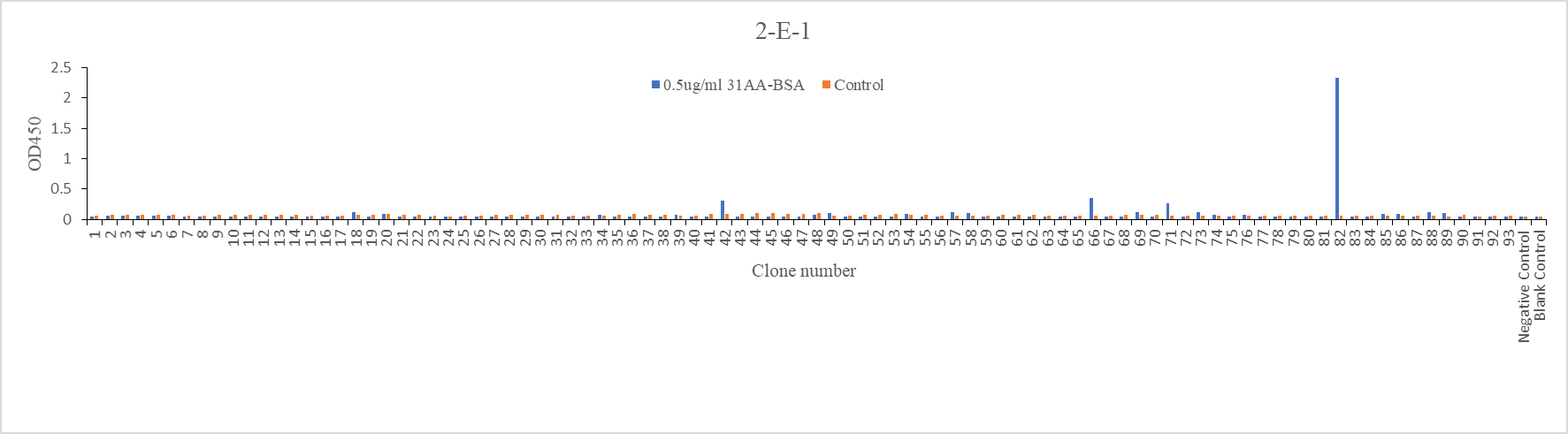


| Clone number | 0.5ug/ml 31AA-BSA | Control |
| --- | --- | --- |
| 94 | 0.056 | 0.063 |
| 95 | 0.06 | 0.07 |
| 96 | 0.06 | 0.073 |
| 97 | 0.065 | 0.076 |
| 98 | 0.058 | 0.081 |
| 99 | 0.264 | 0.077 |
| 100 | 0.054 | 0.059 |
| 101 | 0.056 | 0.063 |
| 102 | 0.053 | 0.055 |
| 103 | 0.051 | 0.053 |
| 104 | 0.05 | 0.052 |
| 105 | 0.05 | 0.052 |
| 106 | 0.051 | 0.053 |
| 107 | 0.05 | 0.05 |
| 108 | 0.051 | 0.051 |
| 109 | 0.051 | 0.051 |
| 110 | 0.053 | 0.057 |
| 111 | 0.412 | 0.052 |
| 112 | 0.049 | 0.049 |
| 113 | 0.048 | 0.127 |
| 114 | 0.049 | 0.051 |
| 115 | 0.076 | 0.048 |
| 116 | 0.075 | 0.05 |
| 117 | 0.049 | 0.053 |
| 118 | 0.102 | 0.06 |
| 119 | 0.504 | 0.052 |
| 120 | 0.068 | 0.049 |
| 121 | 0.074 | 0.142 |
| 122 | 0.048 | 0.049 |
| 123 | 0.047 | 0.103 |
| 124 | 0.051 | 0.05 |
| 125 | 0.052 | 0.055 |
| 126 | 0.053 | 0.053 |
| 127 | 0.049 | 0.051 |
| 128 | 0.049 | 0.051 |
| 129 | 0.049 | 0.055 |
| 130 | 0.052 | 0.048 |
| 131 | 0.049 | 0.05 |
| 132 | 0.051 | 0.058 |
| 133 | 0.053 | 0.056 |
| 134 | 0.052 | 0.062 |
| 135 | 0.068 | 0.05 |
| 136 | 0.049 | 0.049 |
| 137 | 0.048 | 0.054 |
| 138 | 0.049 | 0.054 |
| 139 | 0.049 | 0.052 |
| 140 | 0.05 | 0.058 |
| 141 | 0.084 | 0.054 |
| 142 | 0.05 | 0.051 |
| 143 | 0.047 | 0.05 |
| 144 | 0.067 | 0.049 |
| 145 | 0.187 | 0.05 |
| 146 | 0.048 | 0.051 |
| 147 | 0.049 | 0.051 |
| 148 | 0.049 | 0.054 |
| 149 | 0.05 | 0.055 |
| 150 | 0.05 | 0.052 |
| 151 | 0.048 | 0.052 |
| 152 | 0.048 | 0.049 |
| 153 | 0.048 | 0.048 |
| 154 | 0.048 | 0.048 |
| 155 | 0.047 | 0.047 |
| 156 | 0.048 | 0.049 |
| 157 | 0.074 | 0.055 |
| 158 | 0.052 | 0.051 |
| 159 | 0.072 | 0.052 |
| 160 | 0.047 | 0.049 |
| 161 | 0.048 | 0.051 |
| 162 | 0.046 | 0.047 |
| 163 | 0.047 | 0.048 |
| 164 | 0.048 | 0.051 |
| 165 | 0.051 | 0.055 |
| 166 | 0.049 | 0.052 |
| 167 | 0.083 | 0.065 |
| 168 | 0.074 | 0.048 |
| 169 | 0.048 | 0.048 |
| 170 | 0.048 | 0.049 |
| 171 | 0.048 | 0.048 |
| 172 | 0.05 | 0.049 |
| 173 | 0.049 | 0.05 |
| 174 | 0.132 | 0.05 |
| 175 | 0.186 | 0.05 |
| 176 | 0.05 | 0.116 |
| 177 | 0.048 | 0.051 |
| 178 | 0.049 | 0.05 |
| 179 | 0.047 | 0.05 |
| 180 | 0.049 | 0.049 |
| 181 | 0.081 | 0.053 |
| 182 | 0.134 | 0.053 |
| 183 | 0.071 | 0.052 |
| 184 | 0.115 | 0.053 |
| 185 | 0.05 | 0.05 |
| 186 | 0.053 | 0.054 |
| Negative Control | 0.051 | 0.054 |
| Blank Control | 0.049 | 0.052 |
| Positive Control：0.9750 | | |


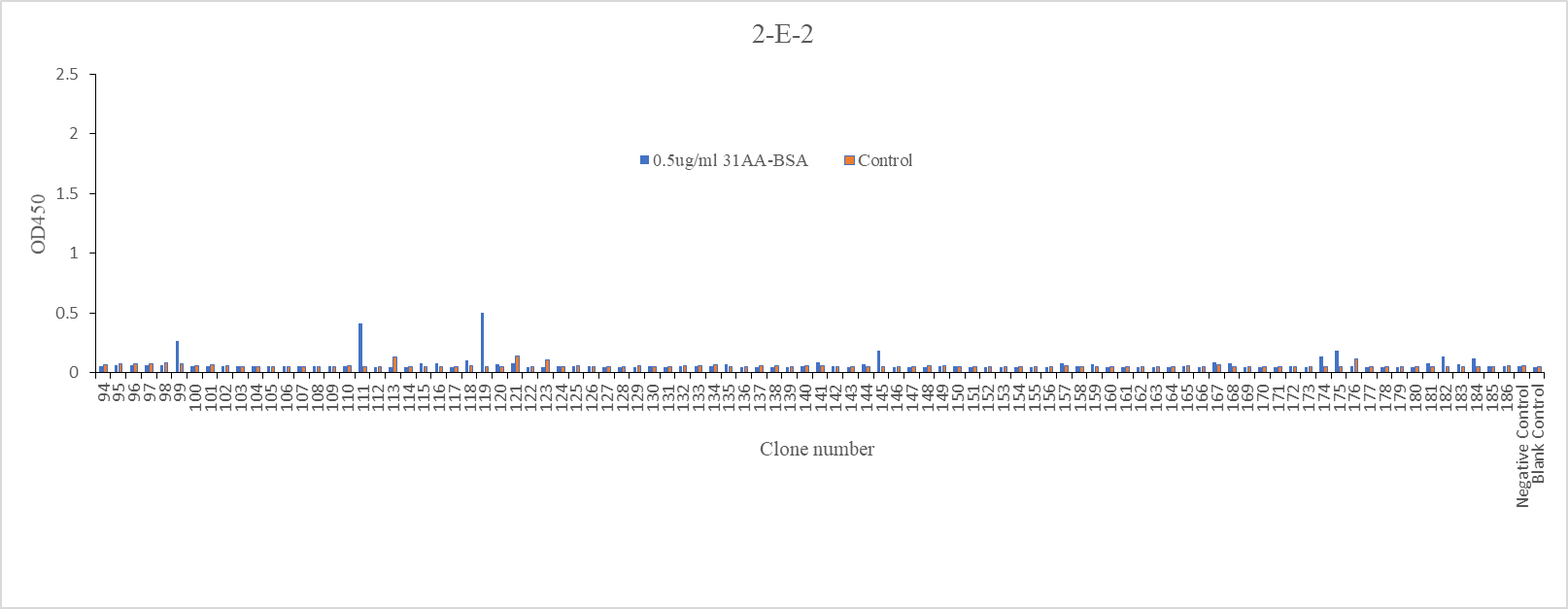


| Clone number | 0.5ug/ml 31AA-BSA | Control |
| --- | --- | --- |
| 187 | 0.05 | 0.111 |
| 188 | 0.061 | 0.107 |
| 189 | 0.054 | 0.073 |
| 190 | 0.065 | 0.097 |
| 191 | 0.062 | 0.08 |
| 192 | 1.833 | 0.097 |
| 193 | 0.063 | 0.081 |
| 194 | 0.066 | 0.105 |
| 195 | 0.051 | 0.1 |
| 196 | 0.05 | 0.111 |
| 197 | 0.049 | 0.104 |
| 198 | 0.049 | 0.123 |
| 199 | 0.051 | 0.109 |
| 200 | 1.347 | 0.103 |
| 201 | 0.062 | 0.088 |
| 202 | 0.05 | 0.096 |
| 203 | 0.053 | 0.119 |
| 204 | 0.05 | 0.131 |
| 205 | 0.05 | 0.106 |
| 206 | 0.049 | 0.117 |
| 207 | 0.049 | 0.104 |
| 208 | 0.05 | 0.095 |
| 209 | 0.051 | 0.072 |
| 210 | 0.05 | 0.072 |
| 211 | 0.053 | 0.131 |
| 212 | 0.049 | 0.144 |
| 213 | 0.051 | 0.137 |
| 214 | 0.049 | 0.128 |
| 215 | 0.049 | 0.112 |
| 216 | 0.049 | 0.156 |
| 217 | 0.051 | 1.397 |
| 218 | 0.05 | 0.061 |
| 219 | 0.054 | 0.129 |
| 220 | 0.049 | 0.129 |
| 221 | 0.049 | 0.136 |
| 222 | 0.048 | 0.126 |
| 223 | 0.049 | 0.103 |
| 224 | 0.049 | 0.076 |
| 225 | 0.051 | 1.785 |
| 226 | 0.05 | 0.065 |
| 227 | 0.054 | 0.099 |
| 228 | 0.049 | 0.102 |
| 229 | 0.049 | 0.113 |
| 230 | 0.049 | 0.12 |
| 231 | 0.05 | 0.093 |
| 232 | 0.048 | 0.083 |
| 233 | 0.051 | 0.075 |
| 234 | 0.055 | 0.079 |
| 235 | 0.053 | 0.081 |
| 236 | 0.049 | 0.089 |
| 237 | 0.049 | 0.084 |
| 238 | 0.048 | 0.102 |
| 239 | 0.048 | 0.098 |
| 240 | 0.048 | 0.081 |
| 241 | 0.049 | 0.081 |
| 242 | 0.048 | 0.079 |
| 243 | 0.052 | 0.085 |
| 244 | 0.047 | 0.077 |
| 245 | 0.049 | 0.076 |
| 246 | 0.047 | 0.091 |
| 247 | 0.047 | 0.089 |
| 248 | 0.047 | 0.079 |
| 249 | 0.049 | 0.071 |
| 250 | 0.05 | 0.084 |
| 251 | 0.053 | 0.083 |
| 252 | 0.049 | 0.07 |
| 253 | 0.048 | 0.084 |
| 254 | 0.054 | 0.086 |
| 255 | 0.048 | 0.079 |
| 256 | 0.048 | 0.079 |
| 257 | 0.049 | 0.068 |
| 258 | 0.048 | 0.075 |
| 259 | 0.052 | 0.081 |
| 260 | 0.05 | 0.071 |
| 261 | 0.05 | 0.067 |
| 262 | 0.049 | 0.085 |
| 263 | 0.049 | 0.078 |
| 264 | 0.048 | 0.072 |
| 265 | 0.049 | 0.068 |
| 266 | 0.049 | 0.078 |
| 267 | 0.051 | 0.07 |
| 268 | 0.049 | 0.075 |
| 269 | 0.05 | 0.091 |
| 270 | 0.049 | 0.09 |
| 271 | 0.051 | 0.087 |
| 272 | 0.048 | 0.068 |
| 273 | 0.051 | 0.069 |
| 274 | 0.049 | 0.081 |
| 275 | 0.052 | 0.089 |
| 276 | 0.049 | 0.079 |
| 277 | 0.051 | 0.08 |
| 278 | 0.05 | 0.097 |
| 279 | 0.049 | 0.097 |
| Negative Control | 0.05 | 0.065 |
| Blank Control | 0.053 | 0.055 |
| Positive Control：1.5180 | | |


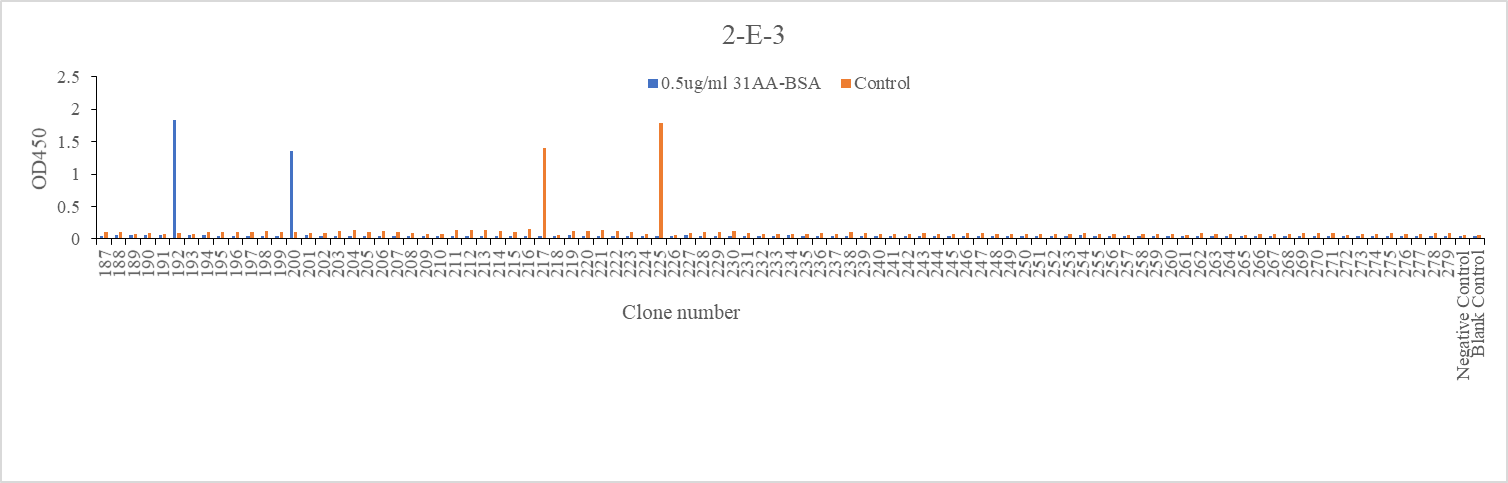


| Clone number | 0.5ug/ml 31AA-BSA | Control |
| --- | --- | --- |
| 280 | 0.069 | 0.061 |
| 281 | 0.069 | 0.063 |
| 282 | 0.064 | 0.06 |
| 283 | 0.091 | 0.065 |
| 284 | 0.113 | 0.062 |
| 285 | 0.076 | 0.068 |
| 286 | 0.05 | 0.064 |
| 287 | 0.057 | 0.071 |
| 288 | 0.178 | 0.057 |
| 289 | 0.103 | 0.054 |
| 290 | 0.132 | 0.053 |
| 291 | 0.103 | 0.055 |
| 292 | 0.113 | 0.063 |
| 293 | 0.351 | 0.052 |
| 294 | 0.105 | 0.053 |
| 295 | 0.06 | 0.05 |
| 296 | 0.082 | 0.057 |
| 297 | 0.15 | 0.05 |
| 298 | 0.127 | 0.05 |
| 299 | 0.095 | 0.05 |
| 300 | 0.538 | 0.05 |
| 301 | 0.281 | 0.05 |
| 302 | 0.127 | 0.05 |
| 303 | 0.039 | 0.049 |
| 304 | 0.074 | 0.059 |
| 305 | 0.115 | 0.051 |
| 306 | 0.088 | 0.05 |
| 307 | 0.133 | 0.048 |
| 308 | 0.049 | 0.049 |
| 309 | 0.285 | 0.048 |
| 310 | 0.053 | 0.05 |
| 311 | 0.044 | 0.048 |
| 312 | 0.073 | 0.063 |
| 313 | 0.34 | 0.05 |
| 314 | 0.057 | 0.06 |
| 315 | 0.064 | 0.051 |
| 316 | 0.118 | 0.049 |
| 317 | 0.051 | 0.049 |
| 318 | 0.049 | 0.049 |
| 319 | 0.04 | 0.049 |
| 320 | 0.053 | 0.055 |
| 321 | 0.059 | 0.05 |
| 322 | 0.054 | 0.05 |
| 323 | 0.064 | 0.05 |
| 324 | 0.094 | 0.05 |
| 325 | 0.13 | 0.049 |
| 326 | 0.045 | 0.05 |
| 327 | 0.039 | 0.05 |
| 328 | 0.213 | 0.059 |
| 329 | 0.171 | 0.049 |
| 330 | 0.047 | 0.049 |
| 331 | 0.043 | 0.049 |
| 332 | 0.044 | 0.048 |
| 333 | 0.088 | 0.048 |
| 334 | 0.047 | 0.049 |
| 335 | 0.048 | 0.049 |
| 336 | 0.05 | 0.061 |
| 337 | 0.039 | 0.054 |
| 338 | 0.046 | 0.196 |
| 339 | 0.045 | 0.111 |
| 340 | 0.046 | 0.048 |
| 341 | 0.043 | 0.048 |
| 342 | 0.042 | 0.049 |
| 343 | 0.039 | 0.05 |
| 344 | 0.051 | 0.056 |
| 345 | 0.045 | 0.102 |
| 346 | 0.06 | 0.05 |
| 347 | 0.045 | 0.047 |
| 348 | 0.046 | 0.048 |
| 349 | 0.045 | 0.047 |
| 350 | 0.043 | 0.049 |
| 351 | 0.042 | 0.048 |
| 352 | 0.056 | 0.057 |
| 353 | 0.04 | 0.051 |
| 354 | 0.048 | 0.051 |
| 355 | 0.048 | 0.049 |
| 356 | 0.092 | 0.05 |
| 357 | 0.046 | 0.049 |
| 358 | 0.102 | 0.05 |
| 359 | 0.04 | 0.048 |
| 360 | 0.05 | 0.057 |
| 361 | 0.043 | 0.059 |
| 362 | 0.039 | 0.057 |
| 363 | 0.037 | 0.054 |
| 364 | 0.141 | 0.05 |
| 365 | 0.044 | 0.049 |
| 366 | 0.049 | 0.049 |
| 367 | 0.04 | 0.048 |
| 368 | 0.053 | 0.06 |
| 369 | 0.231 | 0.052 |
| 370 | 0.046 | 0.05 |
| 371 | 0.045 | 0.05 |
| 372 | 0.042 | 0.049 |
| Negative Control | 0.044 | 0.051 |
| Blank Control | 0.043 | 0.048 |
| Positive Control：1.2250 | | |


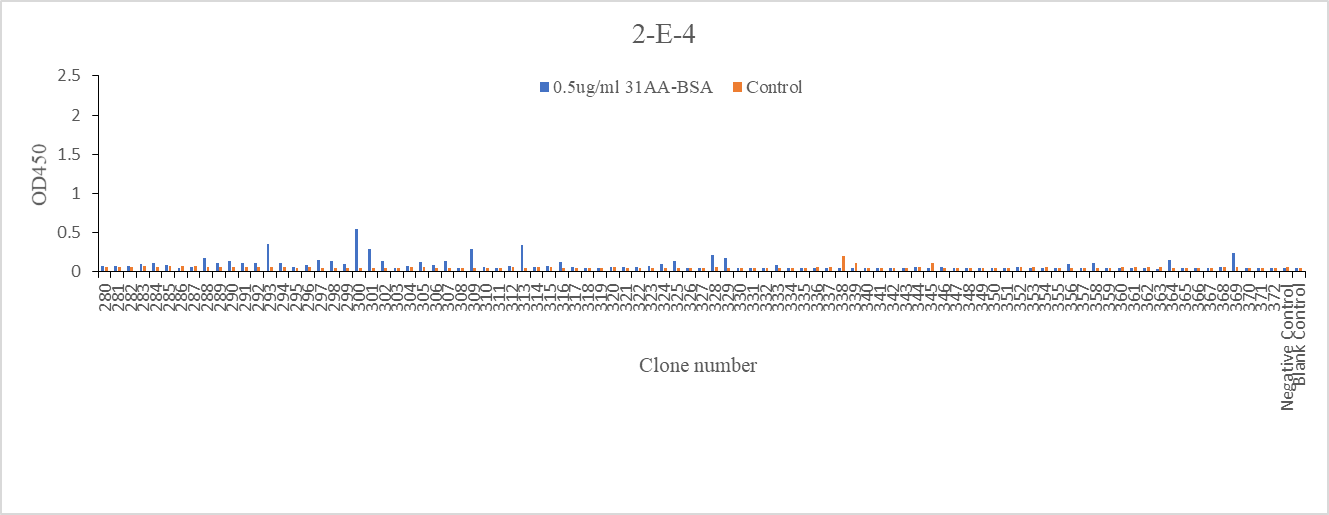


| Clone number | 0.5ug/ml 31AA-BSA | Control |
| --- | --- | --- |
| 373 | 0.137 | 0.056 |
| 374 | 0.11 | 0.058 |
| 375 | 0.066 | 0.056 |
| 376 | 0.107 | 0.061 |
| 377 | 0.098 | 0.056 |
| 378 | 0.057 | 0.057 |
| 379 | 0.104 | 0.057 |
| 380 | 0.053 | 0.061 |
| 381 | 0.117 | 0.055 |
| 382 | 0.074 | 0.05 |
| 383 | 0.076 | 0.05 |
| 384 | 0.088 | 0.051 |
| 385 | 0.069 | 0.053 |
| 386 | 0.071 | 0.049 |
| 387 | 0.068 | 0.051 |
| 388 | 0.071 | 0.058 |
| 389 | 0.101 | 0.053 |
| 390 | 0.105 | 0.057 |
| 391 | 0.05 | 0.049 |
| 392 | 0.072 | 0.049 |
| 393 | 0.101 | 0.049 |
| 394 | 0.078 | 0.048 |
| 395 | 0.078 | 0.051 |
| 396 | 0.077 | 0.052 |
| 397 | 0.097 | 0.058 |
| 398 | 0.051 | 0.052 |
| 399 | 0.074 | 0.051 |
| 400 | 0.066 | 0.048 |
| 401 | 0.05 | 0.048 |
| 402 | 0.086 | 0.048 |
| 403 | 0.054 | 0.051 |
| 404 | 0.051 | 0.052 |
| 405 | 0.119 | 0.061 |
| 406 | 0.078 | 0.049 |
| 407 | 0.081 | 0.048 |
| 408 | 0.083 | 0.05 |
| 409 | 0.067 | 0.048 |
| 410 | 0.083 | 0.048 |
| 411 | 0.08 | 0.05 |
| 412 | 0.057 | 0.054 |
| 413 | 0.052 | 0.057 |
| 414 | 0.089 | 0.053 |
| 415 | 0.079 | 0.055 |
| 416 | 0.082 | 0.05 |
| 417 | 0.052 | 0.049 |
| 418 | 0.084 | 0.049 |
| 419 | 0.053 | 0.05 |
| 420 | 0.093 | 0.053 |
| 421 | 0.094 | 0.088 |
| 422 | 0.083 | 0.047 |
| 423 | 0.082 | 0.05 |
| 424 | 0.051 | 0.058 |
| 425 | 0.082 | 0.047 |
| 426 | 0.084 | 0.048 |
| 427 | 0.085 | 0.05 |
| 428 | 0.087 | 0.063 |
| 429 | 0.102 | 0.174 |
| 430 | 0.072 | 0.049 |
| 431 | 0.081 | 0.051 |
| 432 | 0.087 | 0.049 |
| 433 | 0.05 | 0.059 |
| 434 | 0.07 | 0.048 |
| 435 | 0.062 | 0.052 |
| 436 | 0.071 | 0.056 |
| 437 | 0.111 | 0.059 |
| 438 | 0.084 | 0.05 |
| 439 | 0.089 | 0.055 |
| 440 | 0.095 | 0.05 |
| 441 | 0.082 | 0.049 |
| 442 | 0.09 | 0.048 |
| 443 | 0.08 | 0.05 |
| 444 | 0.056 | 0.054 |
| 445 | 0.181 | 0.056 |
| 446 | 0.05 | 0.051 |
| 447 | 0.094 | 0.052 |
| 448 | 0.053 | 0.049 |
| 449 | 0.049 | 0.049 |
| 450 | 0.05 | 0.05 |
| 451 | 0.048 | 0.051 |
| 452 | 0.051 | 0.056 |
| 453 | 0.05 | 0.054 |
| 454 | 0.079 | 0.048 |
| 455 | 0.06 | 0.051 |
| 456 | 0.078 | 0.049 |
| 457 | 0.05 | 0.05 |
| 458 | 0.049 | 0.048 |
| 459 | 0.09 | 0.053 |
| 460 | 0.056 | 0.056 |
| 461 | 0.05 | 0.058 |
| 462 | 0.051 | 0.051 |
| 463 | 0.054 | 0.053 |
| 464 | 0.05 | 0.052 |
| 465 | 0.068 | 0.055 |
| Negative Control | 0.052 | 0.056 |
| Blank Control | 0.049 | 0.055 |
| Positive Control：1.1830 | | |


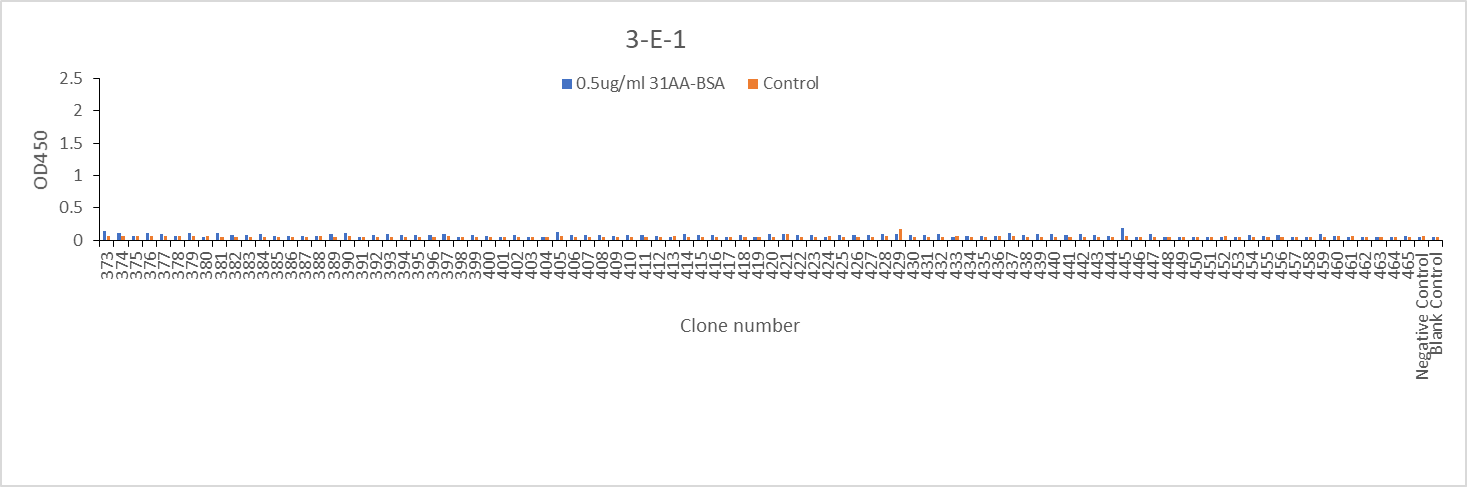


| Clone number | 0.5ug/ml 31AA-BSA | Control |
| --- | --- | --- |
| 466 | 0.102 | 0.051 |
| 467 | 0.068 | 0.05 |
| 468 | 0.089 | 0.053 |
| 469 | 0.087 | 0.057 |
| 470 | 0.079 | 0.054 |
| 471 | 0.077 | 0.057 |
| 472 | 0.078 | 0.059 |
| 473 | 0.106 | 0.122 |
| 474 | 0.083 | 0.053 |
| 475 | 0.061 | 0.049 |
| 476 | 0.061 | 0.053 |
| 477 | 0.066 | 0.05 |
| 478 | 0.074 | 0.049 |
| 479 | 0.064 | 0.049 |
| 480 | 0.063 | 0.05 |
| 481 | 0.051 | 0.055 |
| 482 | 0.085 | 0.055 |
| 483 | 0.054 | 0.053 |
| 484 | 0.078 | 0.051 |
| 485 | 0.062 | 0.048 |
| 486 | 0.059 | 0.049 |
| 487 | 0.076 | 0.048 |
| 488 | 0.24 | 0.049 |
| 489 | 0.063 | 0.049 |
| 490 | 0.062 | 0.051 |
| 491 | 0.074 | 0.064 |
| 492 | 0.074 | 0.156 |
| 493 | 0.059 | 0.051 |
| 494 | 0.06 | 0.068 |
| 495 | 0.064 | 0.05 |
| 496 | 1.267 | 0.049 |
| 497 | 0.309 | 0.058 |
| 498 | 0.084 | 0.076 |
| 499 | 0.058 | 0.059 |
| 500 | 0.315 | 0.054 |
| 501 | 0.068 | 0.049 |
| 502 | 0.067 | 0.051 |
| 503 | 0.09 | 0.05 |
| 504 | 0.075 | 0.051 |
| 505 | 0.068 | 0.072 |
| 506 | 0.082 | 0.052 |
| 507 | 0.058 | 0.049 |
| 508 | 0.943 | 0.051 |
| 509 | 0.063 | 0.051 |
| 510 | 0.07 | 0.049 |
| 511 | 0.083 | 0.049 |
| 512 | 0.063 | 0.051 |
| 513 | 0.051 | 0.053 |
| 514 | 0.086 | 0.049 |
| 515 | 0.055 | 0.05 |
| 516 | 0.066 | 0.049 |
| 517 | 0.077 | 0.05 |
| 518 | 0.058 | 0.049 |
| 519 | 0.071 | 0.048 |
| 520 | 0.078 | 0.049 |
| 521 | 0.071 | 0.055 |
| 522 | 0.073 | 0.074 |
| 523 | 0.068 | 0.048 |
| 524 | 0.755 | 0.048 |
| 525 | 0.074 | 0.05 |
| 526 | 0.07 | 0.048 |
| 527 | 0.07 | 0.051 |
| 528 | 0.066 | 0.049 |
| 529 | 0.07 | 0.052 |
| 530 | 0.06 | 0.051 |
| 531 | 0.069 | 0.048 |
| 532 | 0.497 | 0.048 |
| 533 | 0.067 | 0.049 |
| 534 | 0.071 | 0.049 |
| 535 | 0.075 | 0.047 |
| 536 | 0.075 | 0.051 |
| 537 | 0.053 | 0.056 |
| 538 | 0.078 | 0.05 |
| 539 | 0.076 | 0.049 |
| 540 | 0.081 | 0.049 |
| 541 | 0.054 | 0.049 |
| 542 | 0.053 | 0.05 |
| 543 | 0.072 | 0.049 |
| 544 | 0.079 | 0.05 |
| 545 | 0.077 | 0.052 |
| 546 | 0.061 | 0.053 |
| 547 | 0.069 | 0.049 |
| 548 | 0.062 | 0.048 |
| 549 | 0.06 | 0.048 |
| 550 | 0.067 | 0.05 |
| 551 | 0.07 | 0.049 |
| 552 | 0.06 | 0.051 |
| 553 | 0.063 | 0.052 |
| 554 | 0.077 | 0.056 |
| 555 | 0.055 | 0.05 |
| 556 | 0.071 | 0.055 |
| 557 | 0.059 | 0.055 |
| 558 | 0.049 | 0.056 |
| Negative Control | 0.071 | 0.057 |
| Blank Control | 0.054 | 0.055 |
| Positive Control：1.1630 | | |


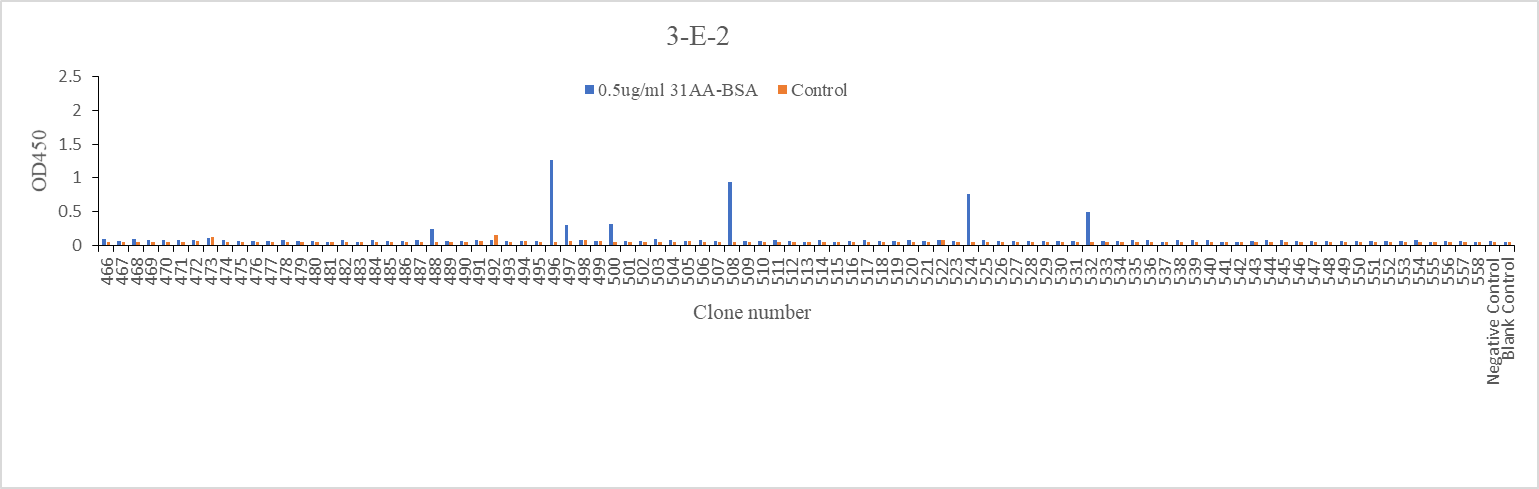


| Clone number | 0.5ug/ml 31AA-BSA | Control |
| --- | --- | --- |
| 559 | 0.12 | 0.058 |
| 560 | 0.161 | 0.062 |
| 561 | 0.116 | 0.55 |
| 562 | 0.057 | 0.063 |
| 563 | 0.059 | 0.062 |
| 564 | 0.059 | 0.062 |
| 565 | 0.132 | 0.059 |
| 566 | 0.13 | 0.079 |
| 567 | 0.093 | 0.057 |
| 568 | 0.08 | 0.061 |
| 569 | 0.05 | 0.06 |
| 570 | 0.08 | 0.064 |
| 571 | 0.05 | 0.066 |
| 572 | 0.05 | 0.06 |
| 573 | 0.054 | 0.065 |
| 574 | 0.092 | 0.077 |
| 575 | 0.124 | 0.055 |
| 576 | 0.127 | 0.064 |
| 577 | 0.117 | 0.053 |
| 578 | 0.1 | 0.057 |
| 579 | 0.083 | 0.064 |
| 580 | 0.077 | 0.067 |
| 581 | 0.084 | 0.073 |
| 582 | 0.06 | 0.071 |
| 583 | 0.06 | 0.054 |
| 584 | 0.051 | 0.059 |
| 585 | 0.533 | 0.066 |
| 586 | 0.049 | 0.064 |
| 587 | 0.106 | 0.065 |
| 588 | 0.105 | 0.06 |
| 589 | 0.12 | 0.062 |
| 590 | 0.101 | 0.073 |
| 591 | 0.141 | 0.055 |
| 592 | 0.049 | 0.055 |
| 593 | 0.905 | 0.064 |
| 594 | 0.072 | 0.051 |
| 595 | 0.075 | 0.052 |
| 596 | 0.045 | 0.05 |
| 597 | 0.089 | 0.053 |
| 598 | 0.166 | 0.054 |
| 599 | 0.2 | 0.064 |
| 600 | 0.107 | 0.061 |
| 601 | 0.051 | 0.056 |
| 602 | 0.094 | 0.069 |
| 603 | 0.089 | 0.067 |
| 604 | 0.088 | 0.062 |
| 605 | 0.052 | 0.07 |
| 606 | 0.069 | 0.062 |
| 607 | 0.169 | 0.061 |
| 608 | 0.049 | 0.071 |
| 609 | 0.049 | 0.061 |
| 610 | 0.098 | 0.062 |
| 611 | 0.136 | 0.066 |
| 612 | 0.104 | 0.064 |
| 613 | 0.05 | 0.068 |
| 614 | 0.094 | 0.06 |
| 615 | 0.106 | 0.068 |
| 616 | 0.113 | 0.067 |
| 617 | 0.079 | 0.064 |
| 618 | 0.104 | 0.066 |
| 619 | 0.1 | 0.058 |
| 620 | 0.047 | 0.07 |
| 621 | 0.05 | 0.061 |
| 622 | 0.107 | 0.069 |
| 623 | 0.129 | 0.066 |
| 624 | 0.048 | 0.066 |
| 625 | 0.048 | 0.064 |
| 626 | 0.048 | 0.071 |
| 627 | 0.096 | 0.073 |
| 628 | 0.086 | 0.064 |
| 629 | 0.091 | 0.072 |
| 630 | 0.104 | 0.079 |
| 631 | 0.161 | 0.062 |
| 632 | 0.108 | 0.058 |
| 633 | 0.049 | 0.061 |
| 634 | 0.101 | 0.074 |
| 635 | 0.057 | 0.073 |
| 636 | 0.089 | 0.068 |
| 637 | 0.109 | 0.072 |
| 638 | 0.116 | 0.077 |
| 639 | 0.057 | 0.066 |
| 640 | 0.048 | 0.066 |
| 641 | 0.05 | 0.073 |
| 642 | 0.133 | 0.076 |
| 643 | 0.094 | 0.073 |
| 644 | 0.086 | 0.064 |
| 645 | 0.14 | 0.06 |
| 646 | 0.051 | 0.075 |
| 647 | 0.175 | 0.065 |
| 648 | 0.189 | 0.063 |
| 649 | 0.158 | 0.063 |
| 650 | 0.048 | 0.064 |
| 651 | 0.12 | 0.061 |
| Negative Control | 0.048 | 0.059 |
| Blank Control | 0.049 | 0.055 |
| Positive Control：1.2250 | | |


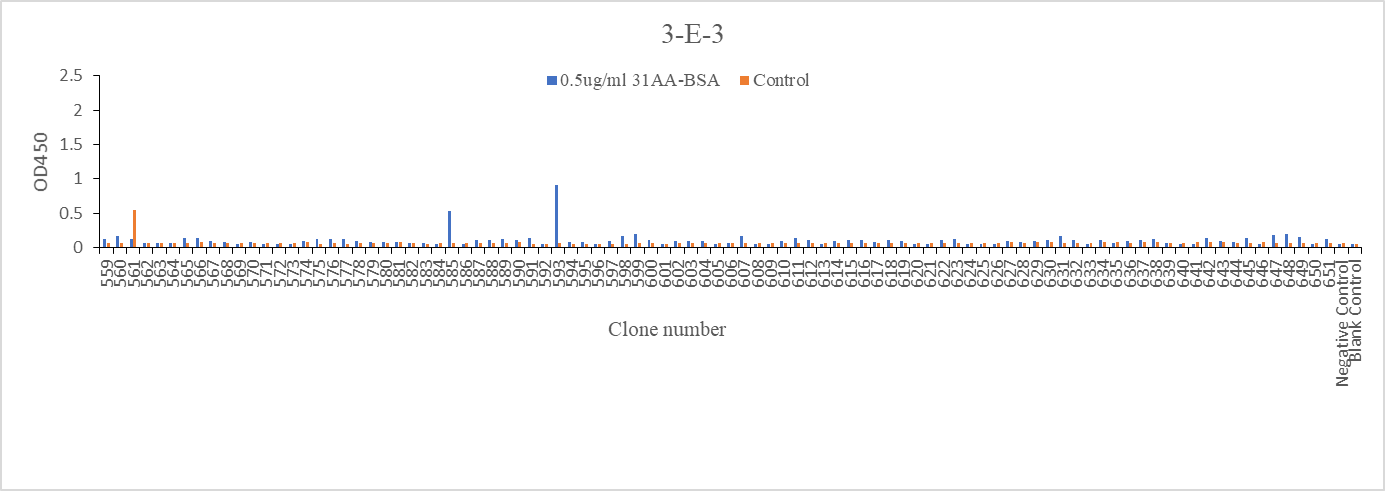


| Clone number | 0.5ug/ml 31AA-BSA | Control |
| --- | --- | --- |
| 652 | 0.12 | 0.054 |
| 653 | 0.142 | 0.053 |
| 654 | 0.102 | 0.051 |
| 655 | 0.118 | 0.053 |
| 656 | 0.105 | 0.056 |
| 657 | 0.102 | 0.057 |
| 658 | 0.054 | 0.05 |
| 659 | 0.067 | 0.06 |
| 660 | 0.05 | 0.054 |
| 661 | 0.095 | 0.052 |
| 662 | 0.079 | 0.05 |
| 663 | 0.092 | 0.052 |
| 664 | 0.072 | 0.062 |
| 665 | 0.061 | 0.061 |
| 666 | 0.103 | 0.05 |
| 667 | 0.061 | 0.053 |
| 668 | 0.082 | 0.054 |
| 669 | 0.127 | 0.05 |
| 670 | 0.136 | 0.05 |
| 671 | 0.083 | 0.051 |
| 672 | 0.049 | 0.052 |
| 673 | 0.073 | 0.052 |
| 674 | 0.097 | 0.049 |
| 675 | 0.058 | 0.055 |
| 676 | 0.16 | 0.049 |
| 677 | 0.048 | 0.061 |
| 678 | 0.745 | 0.229 |
| 679 | 0.047 | 0.048 |
| 680 | 0.074 | 0.054 |
| 681 | 0.078 | 0.049 |
| 682 | 0.08 | 0.054 |
| 683 | 0.051 | 0.061 |
| 684 | 0.153 | 0.049 |
| 685 | 0.047 | 0.048 |
| 686 | 0.048 | 0.282 |
| 687 | 0.088 | 0.05 |
| 688 | 0.079 | 0.049 |
| 689 | 0.048 | 0.049 |
| 690 | 0.08 | 0.051 |
| 691 | 0.059 | 0.057 |
| 692 | 0.051 | 0.05 |
| 693 | 0.093 | 0.055 |
| 694 | 0.084 | 0.049 |
| 695 | 0.082 | 0.05 |
| 696 | 0.048 | 0.05 |
| 697 | 0.049 | 0.049 |
| 698 | 0.083 | 0.058 |
| 699 | 0.06 | 0.058 |
| 700 | 0.113 | 0.05 |
| 701 | 0.05 | 0.048 |
| 702 | 0.048 | 0.048 |
| 703 | 0.049 | 0.057 |
| 704 | 0.048 | 0.048 |
| 705 | 0.081 | 0.048 |
| 706 | 0.069 | 0.052 |
| 707 | 0.058 | 0.055 |
| 708 | 0.048 | 0.048 |
| 709 | 0.089 | 0.047 |
| 710 | 0.075 | 0.052 |
| 711 | 0.084 | 0.049 |
| 712 | 0.048 | 0.048 |
| 713 | 0.081 | 0.048 |
| 714 | 0.078 | 0.05 |
| 715 | 0.088 | 0.057 |
| 716 | 0.098 | 0.048 |
| 717 | 0.048 | 0.049 |
| 718 | 0.071 | 0.05 |
| 719 | 0.073 | 0.051 |
| 720 | 0.048 | 0.051 |
| 721 | 0.068 | 0.047 |
| 722 | 0.065 | 0.053 |
| 723 | 0.053 | 0.057 |
| 724 | 0.095 | 0.049 |
| 725 | 0.088 | 0.048 |
| 726 | 0.075 | 0.048 |
| 727 | 0.047 | 0.048 |
| 728 | 0.077 | 0.05 |
| 729 | 0.047 | 0.047 |
| 730 | 0.06 | 0.052 |
| 731 | 0.11 | 0.053 |
| 732 | 0.098 | 0.052 |
| 733 | 0.048 | 0.049 |
| 734 | 0.073 | 0.048 |
| 735 | 0.09 | 0.048 |
| 736 | 0.078 | 0.049 |
| 737 | 0.091 | 0.05 |
| 738 | 0.105 | 0.052 |
| 739 | 0.128 | 0.061 |
| 740 | 0.13 | 0.054 |
| 741 | 0.048 | 0.048 |
| 742 | 0.087 | 0.052 |
| 743 | 0.11 | 0.059 |
| 744 | 0.101 | 0.053 |
| Negative Control | 0.052 | 0.056 |
| Blank Control | 0.057 | 0.055 |
| Positive Control：1.0250 | | |


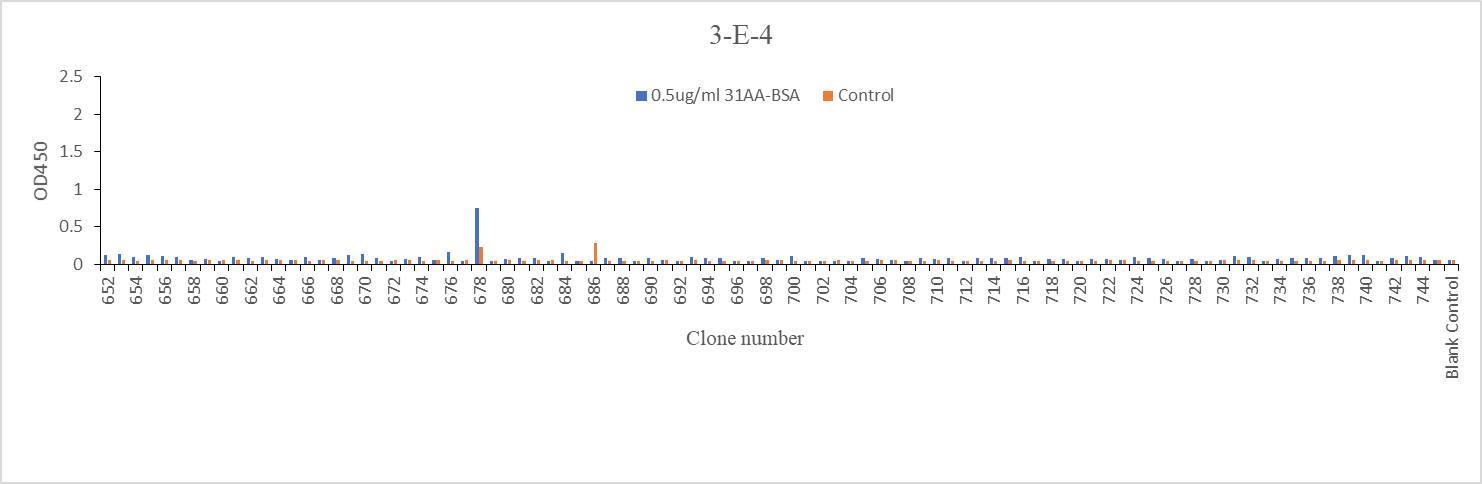

Supplement: Supplementary Materials — Supplementary Figure 1 shows the schematic map of phagemid pSCD-1. Supplementary tables were the results of phage ELISA. [file 7612566.f1.docx]
